# MicroRNAs dysregulated in multiple sclerosis affect the differentiation of CG-4 cells, an oligodendrocyte progenitor cell line

**DOI:** 10.3389/fncel.2024.1336439

**Published:** 2024-02-29

**Authors:** Océane Perdaens, Pauline Bottemanne, Vincent van Pesch

**Affiliations:** ^1^Neurochemistry Group, Institute of NeuroScience, Université catholique de Louvain (UCLouvain), Brussels, Belgium; ^2^Bioanalysis and Pharmacology of Bioactive Lipids, Louvain Drug Research Institute, Université catholique de Louvain (UCLouvain), Brussels, Belgium; ^3^Department of Neurology, Cliniques universitaires Saint-Luc, Université catholique de Louvain (UCLouvain), Brussels, Belgium

**Keywords:** multiple sclerosis, microRNA, miR-33-3p, miR-34c-5p, miR-124-5p, miR-214-3p, oligodendrocyte progenitor cell, oligodendrocyte differentiation

## Abstract

**Introduction:**

Demyelination is one of the hallmarks of multiple sclerosis (MS). While remyelination occurs during the disease, it is incomplete from the start and strongly decreases with its progression, mainly due to the harm to oligodendrocyte progenitor cells (OPCs), causing irreversible neurological deficits and contributing to neurodegeneration. Therapeutic strategies promoting remyelination are still very preliminary and lacking within the current treatment panel for MS.

**Methods:**

In a previous study, we identified 21 microRNAs dysregulated mostly in the CSF of relapsing and/or remitting MS patients. In this study we transfected the mimics/inhibitors of several of these microRNAs separately in an OPC cell line, called CG-4. We aimed (1) to phenotypically characterize their effect on OPC differentiation and (2) to identify corroborating potential mRNA targets via immunocytochemistry, RT-qPCR analysis, RNA sequencing, and Gene Ontology enrichment analysis.

**Results:**

We observed that the majority of 13 transfected microRNA mimics decreased the differentiation of CG-4 cells. We demonstrate, by RNA sequencing and independent RT-qPCR analyses, that miR-33-3p, miR-34c-5p, and miR-124-5p arrest OPC differentiation at a late progenitor stage and miR-145-5p at a premyelinating stage as evidenced by the downregulation of premyelinating oligodendrocyte (OL) [*Tcf7l2*, *Cnp* (except for miR-145-5p)] and mature OL (*Plp1*, *Mbp*, and *Mobp*) markers, whereas only miR-214-3p promotes OPC differentiation. We further propose a comprehensive exploration of their change in cell fate through Gene Ontology enrichment analysis. We finally confirm by RT-qPCR analyses the downregulation of several predicted mRNA targets for each microRNA that possibly support their effect on OPC differentiation by very distinctive mechanisms, of which some are still unexplored in OPC/OL physiology.

**Conclusion:**

miR-33-3p, miR-34c-5p, and miR-124-5p arrest OPC differentiation at a late progenitor stage and miR-145-5p at a premyelinating stage, whereas miR-214-3p promotes the differentiation of CG-4 cells. We propose several potential mRNA targets and hypothetical mechanisms by which each microRNA exerts its effect. We hereby open new perspectives in the research on OPC differentiation and the pathophysiology of demyelination/remyelination, and possibly even in the search for new remyelinating therapeutic strategies in the scope of MS.

## 1 Introduction

Multiple sclerosis (MS) is a chronic inflammatory demyelinating disorder of the central nervous system. Although remyelination occurs, mainly during the early phase of the disease, it remains incomplete and even declines with disease duration and aging, underpinning neurodegeneration (Dulamea, 2017; Yeung et al., 2019).

The myelin sheath is a lipid membrane formed by mature oligodendrocytes (OLs) that spirally wrap around axons to facilitate neuronal conduction and protect axonal integrity (Simons and Nave, 2016). OLs originate from oligodendrocyte progenitor cells (OPCs). The adult brain has a pool of OPCs in the subventricular zone as well as in the surrounding intact gray and white matter that can migrate to the site of myelin turnover and differentiate into mature OLs (Miller, 2002; Hughes et al., 2013; Young et al., 2013; Perlman et al., 2020; Donega et al., 2022). However, in MS, over time OPCs are depleted and fail to migrate and/or differentiate properly as demonstrated by the presence of fewer and often undifferentiating OPCs alongside the loss of mature OLs and the apparent unreceptiveness of axons to remyelination within chronic MS lesions (Wolswijk, 2000; Chang et al., 2002; Kuhlmann et al., 2008; Fancy et al., 2011; Tepavčević and Lubetzki, 2022). Moreover, the myelin sheath formed by remyelination is thinner and extends over a shorter internode distance (Prineas et al., 1993). Demyelination or improper remyelination results in neuro-axonal degeneration, which in turn leads to brain and spinal cord atrophy (Wang C. et al., 2019). Additionally, ongoing demyelination within chronic active (slowly expanding) lesions, characteristic of smoldering MS, is clinically associated with relapse-free disability worsening and disease progression, a process referred to as progression independent of relapse activity (PIRA) (Cree et al., 2019; Elliott et al., 2019; Kappos et al., 2020). While the number of disease-modifying therapies (DMTs) has gradually increased over the last two decades, all of them target the immune system, and clinical research on remyelination-enhancing agents is still very preliminary (Gingele and Stangel, 2020). So far, there is no DMT directly promoting remyelination, which can slow down neurodegeneration and disease progression (Piehl, 2021).

MicroRNAs are small non-coding RNAs that regulate gene expression post-transcriptionally by binding to a complementary seed of their target messenger RNA (mRNA), mostly located in its 3′ untranslated region (3’UTR) (Bartel, 2004). MicroRNAs play a largely acknowledged role in physiological and pathophysiological processes (Bushati and Cohen, 2007) and are also involved in regulating central nervous system myelination. Dynamic changes in the microRNA expression profile finely tune OPC/OL development (Lau et al., 2008; Letzen et al., 2010; Barca-Mayo and Lu, 2012). miR-219, miR-338, and miR-138 promote early OPC differentiation into OLs by targeting OPC proliferation genes. Yet, while miR-219 plays a supporting role up to the late stage of differentiation, miR-138 delays it (Dugas et al., 2010; Zhao et al., 2010). Transgenic mice with OPC/OL-specific Dicer1 ablation, an indispensable endonuclease in microRNA biogenesis, develop tremor and ataxia due to myelination defects (Bartel, 2004; Dugas et al., 2010; Zhao et al., 2010). miR-219, the strongest induced microRNA in differentiating OLs, can partially rescue Dicer1 ablation and improve both lysolecithin-induced demyelination and experimental autoimmune encephalomyelitis (EAE) (Dugas et al., 2010; Wang et al., 2017). Furthermore, miR-219 and miR-338 have been found to be upregulated in normal-appearing white matter but downregulated in inactive white matter lesions of MS patients, and miR-219 is more often undetectable in the cerebrospinal fluid (CSF) of MS patients than controls (Bruinsma et al., 2017; Teuber-Hanselmann et al., 2020). Finally, microRNA dysregulation has been linked to MS (Martin and Illes, 2014). We have previously found 18 microRNAs dysregulated in the CSF of relapsing and/or remitting MS patients, of which 5 have still not been described in MS so far (miR-15a-3p, miR-33a-3p, miR-34c-5p, miR-124-5p, and miR-297) (Perdaens et al., 2020).

Therefore, we hypothesize that these MS-related microRNAs possibly affect OPC differentiation. We thus aimed to screen the effect of a series of these microRNAs on the differentiation of CG-4 cells, a bipotential OPC cell line. We then focused on the microRNAs exhibiting the strongest effect—miR-33-3p, miR-34c-5p, miR-124-5p, miR-145-5p, and miR-214-3p—to further characterize their action on CG-4 differentiation by staging the transfected cells both at the mRNA and protein level and by phenotyping changes via Gene Ontology enrichment analysis. We finally aimed to identify potential mRNA targets by crossing the data of *in silico* analysis and RNA sequencing, which we confirmed by independent RT-qPCR analyses, and propose several mechanisms supporting their effect on OPC differentiation via their predicted targets.

## 2 Methods

### 2.1 Cell culture

We kindly received the Central Glia-4 (CG-4) cells, a permanent cell line derived from rat bipotential oligodendrocyte-type 2-astrocyte (O-2A) progenitor cells (Louis et al., 1992), and the B104 neuroblastoma cells from Dr. M. Grãos (University of Coimbra, Portugal). For CG-4 cell culture, T175 culture flasks or 6-well plates were coated with poly-L-lysine (P2636, Sigma-Aldrich) at 0.04 mg/mL for 30 min at 37°C and washed twice with autoclaved deionized water and once with sterile Dulbecco’s phosphate buffered saline (DPBS, L0615-500, BioWest). Cells were first plated for 30–60 min in Dulbecco’s Modified Eagle Medium (DMEM, L0101-500, BioWest) containing 100 UI/mL penicillin, 100 μg/mL streptomycin (15140-122, Gibco), and 2.5 μg/mL amphotericin B (15290-018, Gibco) (PSA-DMEM) supplemented with 5% fetal bovine serum (FBS, S15898S181H, Gibco), 5 μg/mL human insulin (I9278, Sigma-Aldrich), 2 mM sodium pyruvate (11360–039, Gibco), and 10 mM Hepes (15630-056, Gibco), called recovery medium. Hereafter, CG-4 cells were cultured in a proliferation medium consisting of PSA-DMEM supplemented with 30% B104 conditioned medium containing the essential mitogens, 50 μg/mL human apo-transferrin (T2036, Sigma-Aldrich), 9.8 ng/mL biotin (B4639, Sigma-Aldrich), 40 ng/mL sodium selenite (S5261, Sigma-Aldrich), 5 μg/mL human insulin, and 10 mM Hepes to allow OPC proliferation (Lourenço et al., 2016). To promote their differentiation into OLs, they were cultured in DMEM containing penicillin-streptomycin and 1% GlutaMAX^™^ supplement (35050-061, Gibco) and supplemented with 5 μg/mL bovine insulin (I6634, Sigma-Aldrich), 50 μg/mL human holo-transferrin (T0665, Sigma-Aldrich), 2% B-27^™^ supplement (080085-SA, Gibco), 0.5% FBS, 0.05 μg/mL recombinant rat ciliary neurotrophic factor (CNTF, 450-50, PeproTech), 1% OL-supplement, and 10 mM Hepes, called differentiation medium (O’Meara et al., 2011).

B104 conditioned medium was recovered from the culture medium of B104 cells cultured in uncoated T175 flasks at a cell density of 15,000 cells/cm^2^ for 24 h in PSA-DMEM-F12 medium (L0090-500, BioWest) supplemented with 10% FBS and for 72 h in PSA-DMEM-F12 medium supplemented with 10 μg/mL human holo-transferrin, 5 ng/mL sodium selenite, 16 μg/mL putrescine dihydrochloride (P5780, Sigma-Aldrich), and 6.3 ng/mL progesterone (P8783, Sigma-Aldrich). The latter medium was recovered and centrifuged at 1000 × g for 10 min. The supernatant was filtered (0.22 μm) with 1 μg/mL phenylmethylsulfonyl fluoride (a protease inhibitor, 36978, Thermo Fisher Scientific) and stored at −20°C upon use (Lourenço et al., 2016).

### 2.2 MicroRNA mimic and inhibitor transfection

#### 2.2.1 Double transfection of microRNA mimics

CG-4 cells were plated (day-1) overnight in the proliferation medium at a cell density of 250,000 cells per well in a pre-coated 6-well plate and were separately transfected on day 0 for 6 h and on day 4 for 48 h following the manufacturer’s recommendations. In brief, the miRCURY LNA microRNA mimic or negative control (miR-NC) (Qiagen, Supplementary Table S1B) was diluted in 50 μL Opti-MEM^™^ reduced serum medium (51985026, Thermo Fisher Scientific) for a final concentration of 40 nM (in culture well) and added on top of 50 μL Opti-MEM^™^ containing 3 μL Lipofectamine^™^ 2000 Transfection Reagent (Thermo Fisher Scientific), mixed gently by reverse pipetting (15×) and incubated at room temperature for 5–10 min. This transfection mix (100 μL) was then added dropwise to 900 μL of proliferation medium for 6 h. After 6 h, the medium was replaced by 1.5 mL of proliferation medium. On day 1, the proliferation medium was replaced by 1.5 mL of differentiation medium, of which two-thirds were refreshed on day 4 before the second transfection, which was processed similarly in 1,000 μL total volume, but after 6 h of transfection, 500 μL of fresh differentiation medium was added for an additional 48 h, leaving the transfection mix in the culture wells. Cells were collected on day 6 for RNA extraction.

#### 2.2.2 Single transfection of microRNA mimics

On day-1, CG-4 cells were plated at a cell density of 75,000 cells on a pre-coated glass coverslip placed in a 24-well plate (for immunocytochemistry analysis) or at a cell density of 250,000 cells per well in a pre-coated 6-well plate (for RT-qPCR analysis) for 1 h in the recovery medium and for approximately 20 h in the proliferation medium. On day 0, the cells were transfected with 40 nM miRCURY LNA microRNA mimic or miR-NC (final concentration in culture well) for 6 h (Qiagen) using Lipofectamine^™^ 2000 according to the manufacturer’s protocol (40 nM mimic/1.2 μL Lipofectamine in 40 μL Opti-MEM^™^ added to 360 μL of proliferation medium in 24-well plate and 40 nM mimic/3 μL Lipofectamine in 100 μL Opti-MEM^™^ added to 900 μL of proliferation medium in 6-well plate). After 6 h, the medium was replaced by the differentiation medium (500 μL and 1.5 mL, respectively), and cells were kept in culture for 48 h.

#### 2.2.3 Sequential transfection of microRNA mimics and inhibitors

Cells were plated similarly in pre-coated 6-well plates (day-1) and transfected on day 0 with 40 nM LNA miRCURY microRNA mimic for 4 h followed by a second transfection without (mock transfection with Lipofectamine^™^ 2000 only) or with 40 nM of the corresponding miRCURY LNA microRNA inhibitor (Qiagen, Supplementary Table S1B) for 4 h (without refreshing the medium) using Lipofectamine^™^ 2000 (40 nM mimic or inhibitor/2 μL Lipofectamine in 50 μL Opti-MEM^™^ added to 900 μL of proliferation medium in 6-well plate). The medium was then replaced by 1.5 mL of differentiation medium to culture the cells for an additional 48 h.

In all transfection protocols, the proliferation and differentiation vehicle control conditions were processed similarly but transfected with Lipofectamine^™^ 2000 alone, without microRNA mimic/inhibitor. The proliferation vehicle control was cultured similarly in the proliferation medium only. For each transfection method, three independent experiments were performed with each condition in triplicate.

For endogenous microRNA analysis, cells were plated at a cell density of 100,000 cells per well in a pre-coated 6-well plate. The cells were cultured for 3 days in total in proliferation and/or differentiation medium following the same timing as mentioned in section 2.2.2 and did not undergo any other treatment. Four independent experiments were performed with each condition in triplicate.

### 2.3 RNA and microRNA extraction and RT-qPCR

The culture medium was removed from the 6-well plates, and the wells were washed with 1 mL DPBS. For RNA extraction, TriPure^™^ Isolation Reagent (Roche) was added to the wells, and the plates were stored for a minimum of 30 min at −80°C to help with the cell lysis. RNA was then extracted following the manufacturer’s protocol except for the precipitation step, the samples were stored for 2 h in isopropanol at −20°C to help with RNA precipitation before being centrifuged. RNA concentration and purity were verified by spectrophotometry (Shimadzu BioSpec-nano Spectrophotometer), and 500 ng RNA was reverse transcribed using iScript^™^ cDNA Synthesis Kit (Bio-Rad). MicroRNAs were extracted with QIAzol lysis reagent (Qiagen) following the miRNeasy Mini Kit protocol with RPE and RWT buffer (Qiagen), but we used Enzymax RNA Mini spin columns (EZCR101, Enzymax) instead. RNA concentration and purity were verified by spectrophotometry and set at an equal concentration of 50 ng/μL for all samples. MicroRNAs were reverse transcribed using miRCURY LNA RT (Qiagen). We performed the real-time quantitative polymerase chain reactions (RT-qPCR) using Takyon^™^ No ROX Probe 2X MasterMix Blue dTTP (Eurogentec) for mRNAs and miRCURY LNA SYBR Green PCR kit (Qiagen) for microRNAs on a RotorGene (Qiagen). Primers are listed in Supplementary Tables S1A,B. The specificity was verified with the melt curves. The relative expression was determined by the 2^−ΔΔCt^ method (with ΔΔCt = ΔCt [target m(i)RNA-reference]_sample_ − mean ΔCt [target m(i)RNA-reference]_differentiation (vehicle) control_) (Livak and Schmittgen, 2001). We used hypoxanthine phosphoribosyltransferase 1 (*Hprt1*) as the reference gene for the mRNAs and miR-103a-3p for the microRNAs.

### 2.4 RNA sequencing

RNA sequencing (by synthesis) and the primary bio-informatic analyses were performed by Novogene United Kingdom. All samples of a single experiment with each condition in triplicate (proliferation and differentiation vehicle control, miR-33-3p, miR-34c-5p, miR-124-5p, miR-145-5p, miR-214-3p, and miR-NC cultured in the differentiation medium) passed the pre-sequencing sample quality control with an RNA integrity number between 9 and 9.4 (Agilent 5400), as well as the post-sequencing data quality control with an average of 98.67% clean reads [98.15–98.93% (min-max)], 97.43% Q20 (96.28–97.97%), and 93.04% Q30 (91.23–94.30%). However, one miR-NC replicate was finally excluded from the analysis as it appeared as an outlier. This sample had the lowest Q20 and Q30 percentage.

For library preparation, mRNA was purified from total RNA using poly-T oligo-attached magnetic beads. After fragmentation, the first cDNA strand was synthesized using random hexamer primers, the second using either dUTP for the directional library or dTTP for the non-directional library, followed by end repair, A-tailing, adapter ligation, size selection, USER enzyme digestion (for the directional library only), amplification, and purification. The libraries, quantified with Qubit and real-time PCR and verified by bioanalyzer for size distribution, were pooled and sequenced on Illumina NovaSeq 6000 to generate 150 bp paired-end reads. Twelve gigabytes of raw data (fastq format) were yielded per sample and processed through the fastp software. Clean reads were obtained by removing low-quality reads and reads containing adapter or poly-N. Paired-end clean reads were aligned to the improved rat reference genome (mRatBN7.2) using HISAT2 v2.0.5. The mapped reads of each sample were assembled by StringTie (v1.3.3b), and the number of reads mapped to each gene was counted with featureCounts v1.5.0-p3. Fragments per kilobase million (FPKM) of each gene were calculated based on the length of the gene and the reads count mapped to this gene.

Differential expression analysis between two conditions was performed using the DESeq2 R package (1.20.0). Gene Ontology (GO) enrichment analysis of differentially expressed genes was implemented with the cluster Profiler R package, with correction of gene length bias. The *p*-values were corrected for the false discovery rate by Benjamini and Hochberg’s approach. Differentially expressed genes (DEG) and enriched GO terms were significant if the adjusted *p*-value was ≤0.05.

### 2.5 Immunocytochemistry

Cells were fixed the first time with 4% homemade paraformaldehyde (PFA; 1.04005.1000, Merck) while still in the culture medium (1:2 volumes) for 10 min. The medium was discarded, and cells were fixed a second time in plain 4% PFA at room temperature for 10 min. There were three staining conditions: (1) surface staining of O4 (hybridoma supernatant, AK3203/01, *In Vivo* BioTech) or A2B5 (hybridoma supernatant, CRL-1520, ATCC); (2) intracellular staining of Gfap (ab16997, Abcam) and Mbp (ab7349, Abcam); and (3) intranuclear staining of Pcna (2586, Cell Signaling Technology). For the surface staining of O4 (dilution 1/15) and A2B5 (dilution 1/25), cells were first blocked for 1 h with 5% bovine serum albumin (BSA; A7906, Sigma-Aldrich), then incubated for 1 h with the primary antibody and for 2 h with the secondary antibody [dilution 1/1000; goat anti-mouse IgM Alexa Fluor (AF) 488 (A21042, Thermo Fisher Scientific) and AF647 (A21238, Thermo Fisher Scientific) respectively] in 1% BSA. Then, cells were fixed again. For the intracellular staining of Gfap (dilution 1/500) and Mbp (dilution 1/500) or the intranuclear staining of Pcna (dilution 1/2400) separately, cells were blocked and permeabilized with 0.3% Triton X-100 (T8787, Sigma-Aldrich) and 5% BSA for 1 h. Moreover, for Pcna, this step was preceded by a permeabilization with 100% methanol for 10 min at −20°C. Cells were then incubated overnight at 4°C with the primary antibodies and 2h at room temperature with the secondary antibodies (dilution 1/1000; AF488 goat anti-rabbit IgG (A-11008, Thermo Fisher Scientific) and AF555 goat anti-rat IgG (A21434, Thermo Fisher Scientific), or AF488 goat anti-mouse IgG (A-11001, Thermo Fisher Scientific), respectively) in 0.3% Triton X-100 and 1% BSA. Then, the coverslips were mounted on VWR^®^ Superfrost^®^ Plus Micro Slide using ProLong^™^ Gold Antifade Mountant with DAPI (Thermo Fisher Scientific). All buffers were prepared with DPBS containing calcium and magnesium (14080-055, Gibco). All steps were performed at room temperature, except the overnight incubation, and cells were washed three times in between steps, except once after methanol permeabilization and not after blocking. The mounted coverslips were scanned by the Zeiss Axio Scan.z1, and the QuPath open-source software (version 0.4.0.) was trained to count distinctively the marked cells, except for Gfap (Bankhead et al., 2017). Gfap expression was estimated to the total number of cells by visualizing grid-wise each coverslip and scored as 0 = no Gfap-positive (Gfap^+^) cells, 1 = a few Gfap^+^ cells scattered over the whole coverslip, 2 = a few Gfap^+^ cells congregated in a few grids of the coverslip, 3 = a few Gfap^+^ cells in several grids of the coverslip, 4 = many Gfap^+^ cells in several grids of the coverslip, and 5 = many Gfap^+^ cells over the whole coverslip. The evaluator was blinded to the experimental conditions.

Each staining was performed on a single replicate per condition within each experiment, repeated three to five times. The specificity of the primary antibodies was verified by staining a differentiation vehicle control condition with the secondary antibody/antibodies only, correspondingly to each staining condition within each experiment.

### 2.6 *In silico* analysis

We interrogated 4 databases for the predicted mRNA targets of each investigated microRNA separately: miRDB,^1^ TargetScanHuman 8.0,^2^ DIANA-microT-CDS,^3^ and miRWalk 3^4^ (Paraskevopoulou et al., 2013; Sticht et al., 2018; McGeary et al., 2019; Chen and Wang, 2020). We preselected targets predicted by at least three databases for both *Rattus norvegicus* and *Homo sapiens* and also found downregulated by the RNA sequencing analysis as compared to the differentiation vehicle control and miR-NC conditions. We checked using a PubMed search (“*mRNA-target name* + oligodendrocyte”) if these were involved accordingly in OPC differentiation, i.e., if their downregulation could explain the effect of the microRNA, or checked if they were listed in the gene annotations of downregulated Gene Ontology/Biological Process terms of interest.

### 2.7 Statistical analysis

We used GraphPad Prism 8.0.2 for all statistical analyses. Outliers identified by Grubb’s test were excluded (max 1/condition). Normality was verified by the D’Agostino & Pearson test, and a parametric (ordinary or Brown–Forsythe in case of significantly different standard deviations) or a non-parametric (Kruskal–Wallis) one-way ANOVA was performed accordingly. Dunnett’s (parametric analysis) or Dunn’s (non-parametric analysis) multiple comparison post-test was used to compare all conditions to the differentiation vehicle control or the differentiation miR-NC, Sidak’s, Tamhane’s T2, or Dunn’s multiple comparisons post-test was used as recommended to compare the mimic condition to its inhibitor. A non-parametric Mann–Whitney *U*-test was performed for the statistical analysis of the relative expression of endogenous microRNAs between the proliferation and differentiation control conditions. For plots built in R, we used the packages “cowplot,” “gplots,” “ggplot2,” “grid,” “gridExtra,” “patchwork,” and “stringr.”

## 3 Results

### 3.1 microRNA mimics affect the differentiation of CG-4 cells

**Figure 1:**
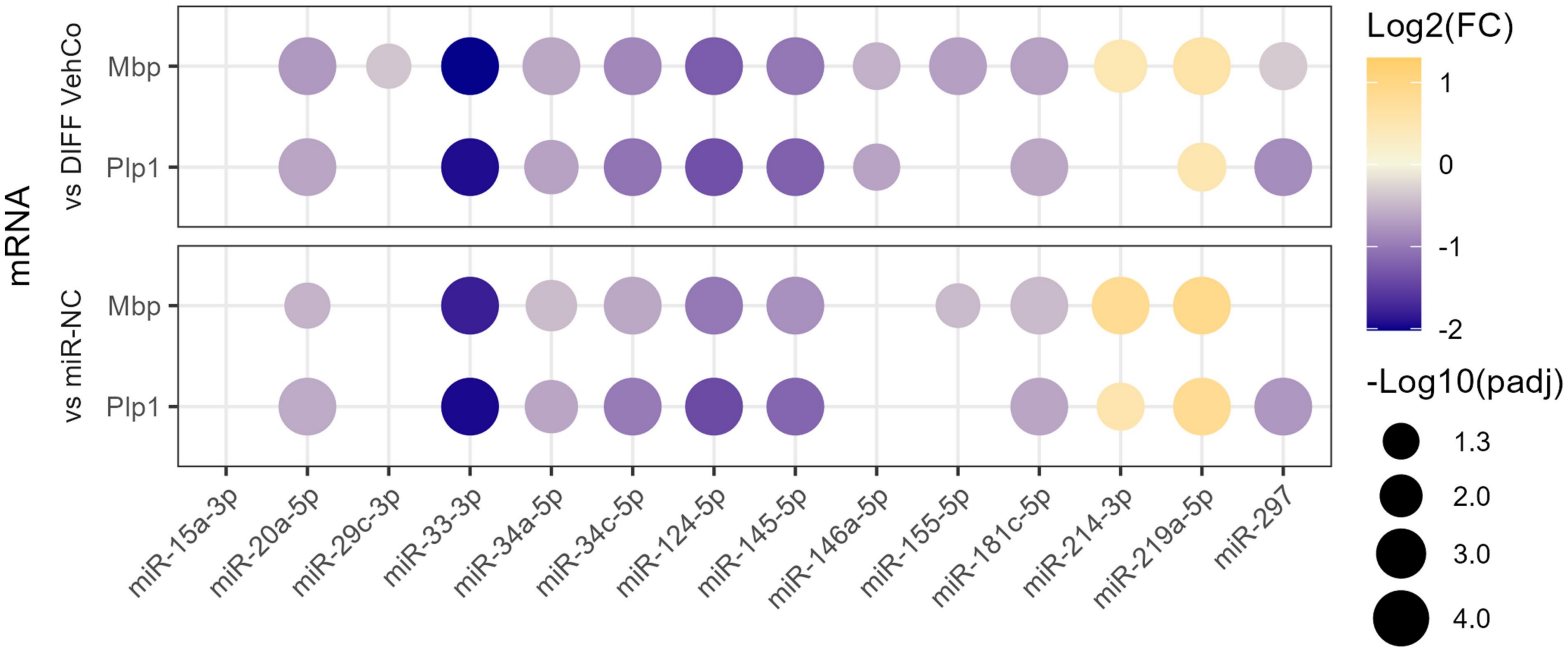
The effect of 13 microRNA mimics on the differentiation of CG-4 cells in RT-qPCR (screening). This dot plot shows the fold change (FC) of the relative expression of the differentiation markers *Mbp* and *Plp1* to the differentiation vehicle control (DIFF VehCo) and the microRNA mimic negative control (miR-NC) resulting from the double transfection of each microRNA mimic, as labeled on the *x*-axis, in CG-4 cells in differentiation culture conditions. The color of each dot represents the fold change (expressed in logarithm base 2 [Log2(FC)]) of the relative expression, scaled by a blue-to-yellow color gradient from −2 to 1.3 (blue corresponding to a negative fold change, yellow to a positive), and its size represents the adjusted *p*-value (*p*_adj_) (expressed as minus logarithm base 10 [−Log10(*p*_adj_)]) as indicated in the plot legend. The adjusted *p*-value was calculated by Tukey’s multiple comparison test, as recommended, following a parametric one-way ANOVA on each microRNA separately with both controls. Only the significant data are depicted; thus, missing dots correspond to non-significant data. −Log10(*p*_adj_) ≥1.3 corresponds to the significance level of *p* ≤ 0.05, −Log10(*p*_adj_) of 2.0 corresponds to *p* = 0.01, −Log10(*p*_adj_) of 3.0 to *p* = 0.001, and −Log10(*p*_adj_) of 4.0 to *p* = 0.0001. Data were from three experiments for each microRNA with each condition in triplicate. The corresponding bar plots showing both the statistically significant and non-significant results can be found in Supplementary Figure S1.

We have previously identified 21 microRNAs dysregulated in the cerebrospinal fluid, serum, and/or peripheral blood mononuclear cells of relapsing and/or remitting MS patients compared to symptomatic/healthy controls (Perdaens et al., 2020). Therefore, we aimed to investigate whether these microRNAs could affect the differentiation of OPCs using the CG-4 cell line. The microRNAs were chosen based on the strength of their dysregulation or their uniqueness in the setting of MS. As a preliminary screening, CG-4 cells were transfected on day 0 and day 4 separately with one of the 13 microRNA mimics (miR-15a-3p, miR-20a-5p, miR-29c-3p, miR-33-3p, miR-34a-5p, miR-34c-5p, miR-124-5p, miR-145-5p, miR-146a-5p, miR-155-5p, miR-181c-5p, miR-214-3p, and miR-297) and cultured in the differentiation medium from day 1 until day 6. We used miR-219a-5p as a positive control of differentiation, as well as the negative control microRNA mimic (miR-NC). Most of these microRNAs significantly reduced the expression of the differentiation markers, myelin basic protein (*Mbp*), and proteolipid protein 1 (*Plp1*) at the mRNA level. miR-214-3p was the only microRNA that increased their expression but to a lower extent than miR-219a-5p (Figure 1; Supplementary Figure S1).

**Figure 2:**
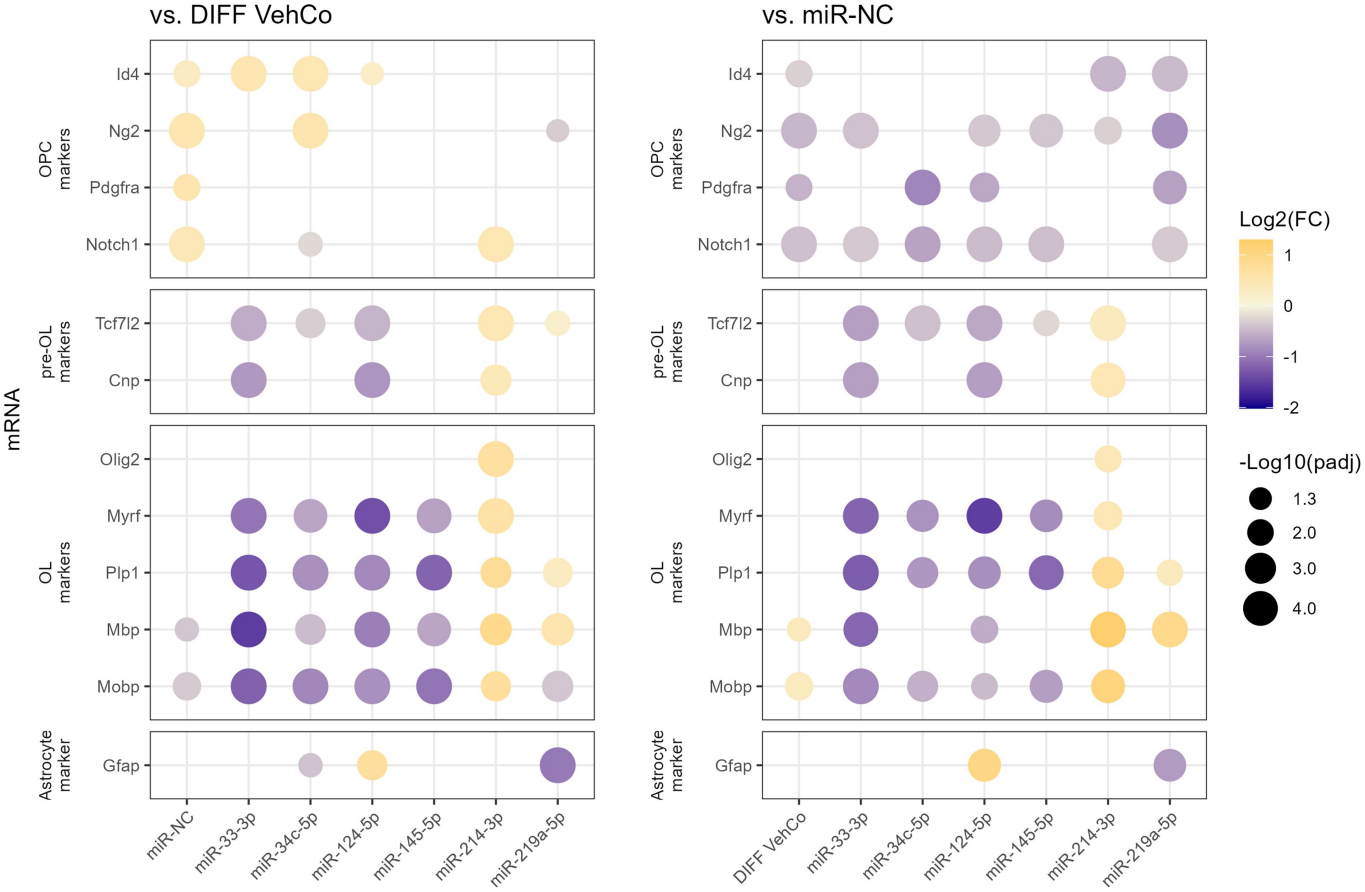
The effect of five microRNA mimics on the differentiation of CG-4 cells in RT-qPCR (confirmation). This dot plot shows the fold change (FC) of the relative expression of several OPC, pre-OL, OL, and astrocyte markers, as labeled on the *y*-axis, to the differentiation vehicle control (DIFF VehCo) and the microRNA mimic negative control (miR-NC), resulting from the single transfection of each microRNA mimic, as labeled on the *x*-axis, in CG-4 cells in differentiation culture conditions. The color of each dot represents the fold change (expressed in logarithm base 2 [Log2(FC)]) of the relative expression, scaled by a blue-to-yellow color gradient from −2 to 1.3 (blue corresponding to a negative fold change, yellow to a positive), and its size represents the adjusted *p*-value (*p*_adj_) (expressed as minus logarithm base 10 [−Log10(*p*_adj_)]) as indicated in the plot legend. The adjusted *p*-value was calculated by Dunn’s or Dunnett’s multiple comparison test to each control separately, i.e., the differentiation vehicle control and miR-NC, as recommended following a parametric/non-parametric one-way ANOVA on all conditions, including the proliferation vehicle control (not shown). Only the significant data are depicted; thus, missing dots correspond to non-significant data. −Log10(*p*_adj_) ≥ 1.3 corresponds to the significance level of *p* ≤ 0.05, −Log10(*p*_adj_) of 2.0 corresponds to *p* = 0.01, −Log10(*p*_adj_) of 3.0 to *p* = 0.001, and −Log10(*p*_adj_) of 4.0 to *p* = 0.0001. Data were from three experiments with each condition in triplicate. OPC, oligodendrocyte progenitor cells; pre-OL, premyelinating oligodendrocytes; OL, oligodendrocytes. The corresponding bar plots showing both the statistically significant and non-significant results can be found in Supplementary Figure S2.

From this point onward, we decided to focus on the microRNA species that reduced the expression of the differentiation markers by at least a factor of 2 compared to the differentiation vehicle control, i.e., miR-33-3p, miR-34c-5p, miR-124-5p, and miR-145-5p. miR-214-3p was also selected as it was the only microRNA significantly increasing the expression of *Plp1* and *Mbp*, although by less than a factor 2. We used a simplified protocol with a single transfection followed by a 48 h culture in the differentiation medium, as this appeared to be sufficient to identify differences in the differentiation stages among the transfected conditions and would simplify further comparisons with the respective microRNA inhibitors. We further aimed to stage each of the transfected cells by measuring the relative expression of several OPC/OL markers (Figure 2; Supplementary Figure S2) (Mitew et al., 2014). miR-33-3p, miR-34c-5p, miR-124-5p, and miR-145-5p significantly downregulated the expression of mature OL markers—myelin regulatory factor (*Myrf*), *Plp1*, *Mbp*, and myelin-associated oligodendrocyte basic protein (*Mobp*)*—*as compared to the differentiation vehicle control and miR-NC, except for *Mbp* with miR-34c-5p and miR-145-5p when compared to miR-NC. miR-33-3p was the strongest inhibitor for all markers except *Myrf*. On the contrary, miR-214-3p promoted CG-4 differentiation, remarkably to an even greater extent than miR-219a-5p, as seen by the significant upregulation of these markers (Figure 2; Supplementary Figure S2). Interestingly, these microRNAs affected the differentiation already at an early premyelinating stage, as seen by the significant downregulation of transcription factor 7 like 2 (*Tcf7l2*) and 2′,3′-cyclic nucleotide 3′phosphodiesterase (*Cnp*) by miR-33-3p and miR-124-5p, and their upregulation by miR-214-3p against both controls, while miR-34c-5p significantly downregulated *Tcf7l2* only. The OPC marker, inhibitor of DNA binding 4 (*Id4*), was significantly upregulated by miR-33-3p, miR-34c-5p, miR-124-5p, and miR-NC as well against the differentiation vehicle control and significantly downregulated by miR-214-3p and miR-219a-5p against miR-NC. Moreover, miR-214-3p significantly increased the expression of oligodendrocyte transcription factor 2 (*Olig2*), which is involved in oligodendroglial specification, highly expressed in myelinating OLs and sufficient to promote OPC differentiation by inducing SRY-box transcription factor 10 (Sox10) and other differentiation genes through chromatin remodelers (Figure 2; Supplementary Figure S2) (Liu et al., 2007; Meijer et al., 2012; Yu et al., 2013). Given the bipotentiality of CG-4 progenitor cells that can differentiate into type-2 astrocytes or mature OLs, depending on the culture medium, we explored this axis as well and found that miR-124-5p increased and miR-219a-5p decreased the expression of glial fibrillary acidic protein (*Gfap*), while cultured in the OL-differentiation medium (Figure 2; Supplementary Figure S2).

**Figure 3:**
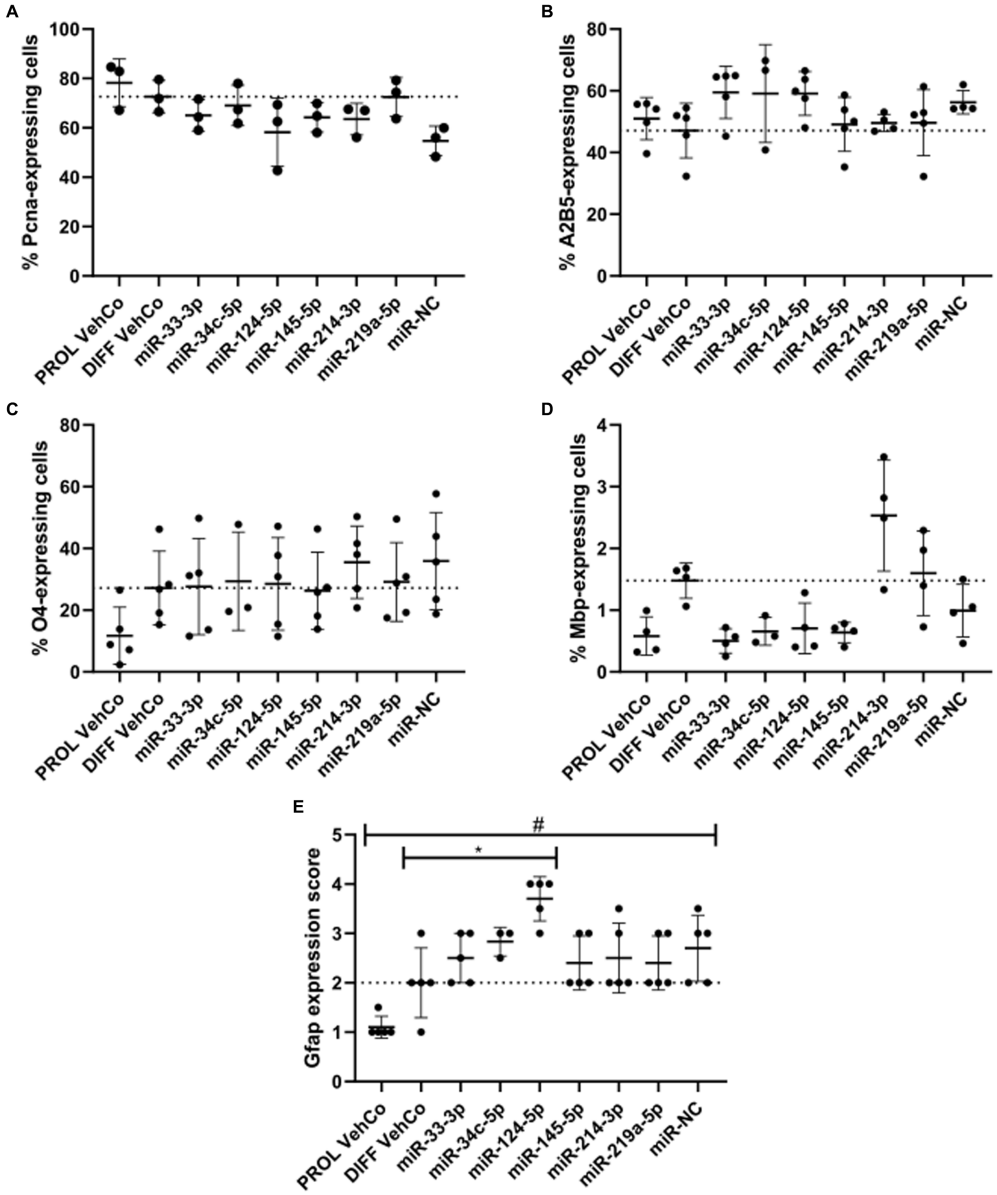
The effect of five microRNA mimics on the differentiation of CG-4 cells in immunocytochemistry. The plots show the percentage of cells expressing **(A)** the proliferation marker Pcna, **(B)** the OPC marker A2B5, **(C)** the early differentiation marker O4, and **(D)** the late differentiation marker Mbp, as well as **(E)** the score of Gfap expression (mean with SD), calculated **(A–D)**/estimated **(E)** to the total number of cells (based on the detection of the nucleus counterstained with DAPI), resulting from the single transfection of each microRNA mimic (as labeled on the *x*-axis) in CG-4 cells in differentiation culture conditions, as well as the proliferation vehicle control (PROL VehCo). The score for Gfap expression ranges from 0 = none to 5 = abundant (namely 0 = no Gfap-positive (Gfap^+^) cells, 1 = a few Gfap^+^ cells on the whole coverslip, 2 = a few Gfap^+^ cells in a few grids of the coverslip, 3 = a few Gfap^+^ cells in several grids of the coverslip, 4 = many Gfap^+^ cells in several grids of the coverslip, 5 = many Gfap^+^ cells on the whole coverslip). The dotted line represents the mean value of the differentiation vehicle control (DIFF VehCo). The non-parametric one-way ANOVAs on all conditions were not significant, except for Gfap: adjusted *p*-value = 0.0148 (*) for miR-124-5p against DIFF VehCo, and adjusted *p*-value = 0.0261 (#) for PROL VehCo against microRNA mimic negative control (miR-NC) on Dunn’s multiple comparison test. Yet, Pcna-expression tended to decrease in miR-NC-transfected cells (non-significant non-parametric one-way ANOVA, but significant Dunn’s post-test against PROL VehCo). Data were from three to five experiments, in single replicates for each experimental and staining condition within each experiment. OPC, oligodendrocyte progenitor cells; SD, standard deviation. Representative fluorescence micrographs can be found in Supplementary Figures S3A–D.

Next, we phenotypically characterized the transfected CG-4 cells at the protein level by immunocytochemistry. First, proliferation was not significantly affected by the transfection of the several microRNA mimics against the proliferation and differentiation vehicle controls as evidenced by proliferating cell nuclear antigen (Pcna), an auxiliary protein regulating DNA replication/repair, cell cycle control, etc., and hence a marker of the early G1 and S phases of the cell cycle (Strzalka and Ziemienowicz, 2011). Yet, it tended to decrease in miR-NC-transfected cells (Figure 3A; Supplementary Figure S3A). Cells transfected with miR-33-3p, miR-34c-5p, and miR-124-5p tended to show a higher proportion of A2B5^+^ cells, a progenitor cell marker, than the differentiation vehicle control. A trend toward a higher proportion of O4^+^ cells, an early differentiation marker, was observed in miR-214-3p-transfected cells (Figures 3B,C; Supplementary Figures S3B,C) (Gard and Pfeiffer, 1990). miR-33-3p, miR-34c-5p, miR-124-5p, and miR-145-5p did not affect O4-positivity as compared to the differentiation vehicle control but tended to decrease the proportion of Mbp-expressing cells, while miR-214-3p tended to increase it (Figures 3C,D; Supplementary Figures S3C,D). The outcomes of the one-way ANOVA did not exhibit any statistically significant disparities. However, it is important to note that the analysis was performed on all conditions collectively, rather than individually, for each microRNA mimic condition versus the controls. In this latter, less stringent analysis, some results were statistically significant (data not shown). Finally, miR-124-5p transfected cells expressed significantly more Gfap, based on a visual score (Figure 3E; Supplementary Figure S3D). Representative fluorescence micrographs can be found in Supplementary Figures S3A–D, whereby Mbp-staining suggests the branching morphology of mature oligodendrocytes.

From these results at the mRNA and protein level, we can thus conclude that miR-33-3p, miR-34c-5p, miR-124-5p, and miR-145-5p impeded to a certain extent the differentiation of CG-4 cells into more mature OLs by retaining the cells within a late progenitor (miR-33-3p, miR-34c-5p, and miR-124-5p)-early premyelinating (miR-145-5p) stage, while miR-214-3p induced their differentiation to a further stage.

### 3.2 microRNA inhibitors antagonize their corresponding exogenous microRNA in differentiating CG-4 cells

**Figure 4:**
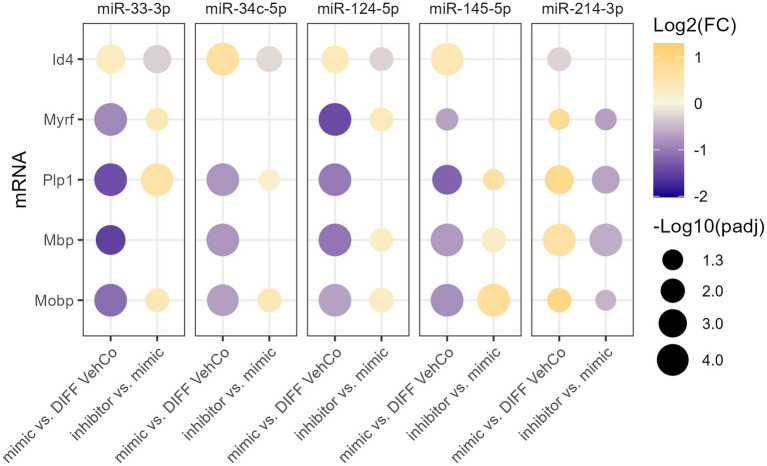
The effect of five microRNA mimics and their inhibitors on the differentiation of CG-4 cells in RT-qPCR. This dot plot shows the fold change (FC) of the relative expression of the OPC marker (*Id4*) and OL markers (*Myrf*, *Plp1*, *Mbp*, and *Mobp*) as labeled on the *y*-axis, resulting from the sequential transfection of each microRNA mimic followed or not by its inhibitor in CG-4 cells in differentiation culture conditions. For each microRNA mimic, the fold change was calculated to the differentiation vehicle control (DIFF VehCo), while for each microRNA inhibitor, it was calculated to its corresponding microRNA mimic, as labeled on the *x*-axis. The color of each dot represents the fold change (expressed in logarithm base 2 [Log2(FC)]) of the relative expression, scaled by a blue-to-yellow color gradient from −2 to 1.3 (blue corresponding to a negative fold change, yellow to a positive), and its size represents the adjusted *p*-value (*p*_adj_) (expressed as minus logarithm base 10 [−Log10(*p*_adj_)]) as indicated in the plot legend. The adjusted *p*-value was calculated by Dunn’s or Dunnett’s multiple comparison test of each condition to the differentiation vehicle control and by Sidak’s, Tamhane’s T2, or Dunn’s multiple comparison test between the mimic and its inhibitor, as recommended following a parametric/non-parametric one-way ANOVA on all conditions, including the proliferation vehicle control (not shown). Only the significant data are depicted; thus, missing dots correspond to non-significant data. −Log10(*p*_adj_) ≥1.3 corresponds to the significance level of *p* ≤ 0.05, −Log10(*p*_adj_) of 2.0 corresponds to *p* = 0.01, −Log10(*p*_adj_) of 3.0 to *p* = 0.001, and −Log10(*p*_adj_) of 4.0 to *p* = 0.0001. Data were from three experiments for each microRNA with each condition in triplicate. OPC, oligodendrocyte progenitor cells; OL, oligodendrocytes. The corresponding bar plots showing both the statistically significant and non-significant results can be found in Supplementary Figure S4.

To verify the specificity of the microRNA mimics, we sought to counter their effect by transfecting the cells first with the microRNA mimic, followed by a second transfection with the respective microRNA inhibitor. This was compared to the transfection of the mimic followed by a mock transfection (without its inhibitor), which yielded, similarly to the single transfection, the expected dysregulation of *Id4*, *Myrf*, *Plp1*, *Mbp*, and *Mobp* for all microRNAs when compared to the differentiation vehicle control, reaching the significance level for all except for miR-34c-5p on *Myrf*. On the other hand, the microRNA inhibitors significantly antagonized the effect of their corresponding exogenous microRNA on the expression of 3 to 4 of these markers, depending on the microRNA species (Figure 4; Supplementary Figure S4).

### 3.3 The endogenous microRNA expression is partially consistent with their effect on CG-4 cell differentiation

**Figure 5:**
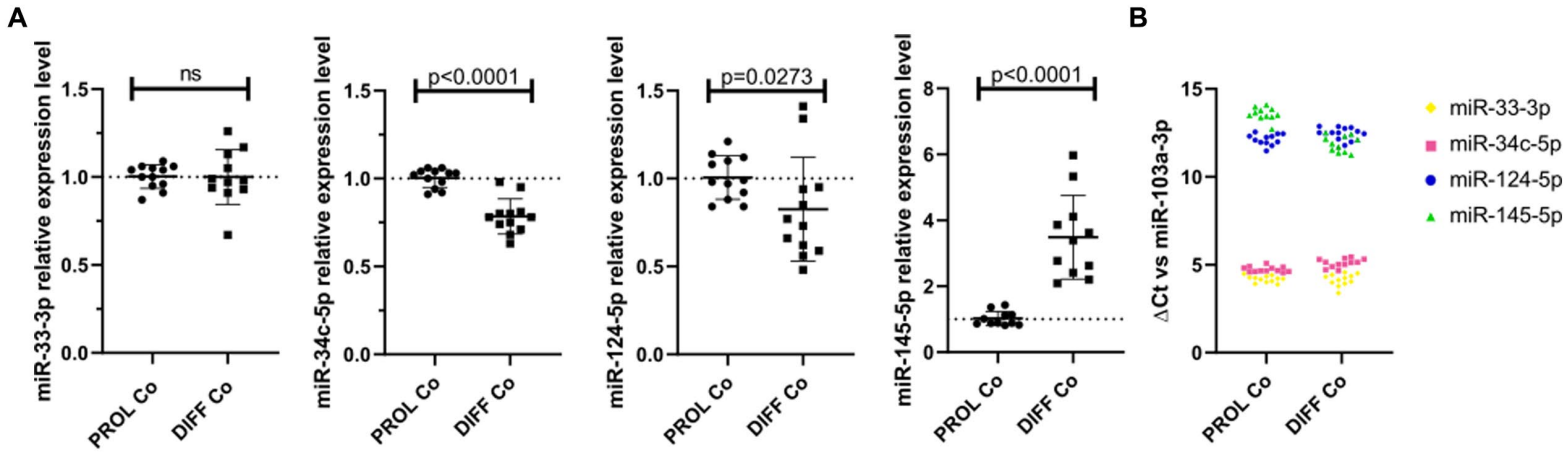
Endogenous microRNAs in proliferating and differentiating CG-4 cells. The plots show **(A)** the relative expression in RT-qPCR (mean with SD of 2^−ΔΔCt^) of each endogenous microRNA in CG-4 cells cultured in the proliferation or differentiation medium, as well as **(B)** the ΔCt of each microRNA with miR-103a-3p as reference (ΔCt = Ct(target microRNA)_sample_ – Ct(reference miR-103a-3p)_sample_), which is representative of the relative abundance of each microRNA. Each color in **(B)** corresponds to a microRNA species, as indicated in the plot legend. The *p*-value (*p*) was calculated by a Mann–Whitney *U*-test, ns for not significant. Data were from four experiments with each condition in triplicate. PROL Co, proliferation control; DIFF Co, differentiation control; SD, standard deviation.

We verified the endogenous microRNA expression in CG-4 cells cultured for 24 h in the proliferation medium, followed by an additional 48 h in either the proliferation or differentiation medium. While miR-33-3p was not significantly dysregulated, miR-34c-5p and miR-124-5p were significantly downregulated in differentiating CG-4 cells. miR-145-5p was upregulated under differentiation culture conditions, which is inconsistent with the observed effect of the microRNA mimic, and miR-214-3p was undetectable in both conditions (Figure 5A). miR-124-5p and miR-145-5p were moreover the least abundant, as evidenced by their higher ΔCt with miR-103a-3p as reference (Figure 5B).

### 3.4 RNA sequencing reveals the changes in cell fate directed by each microRNA

**Figure 6:**
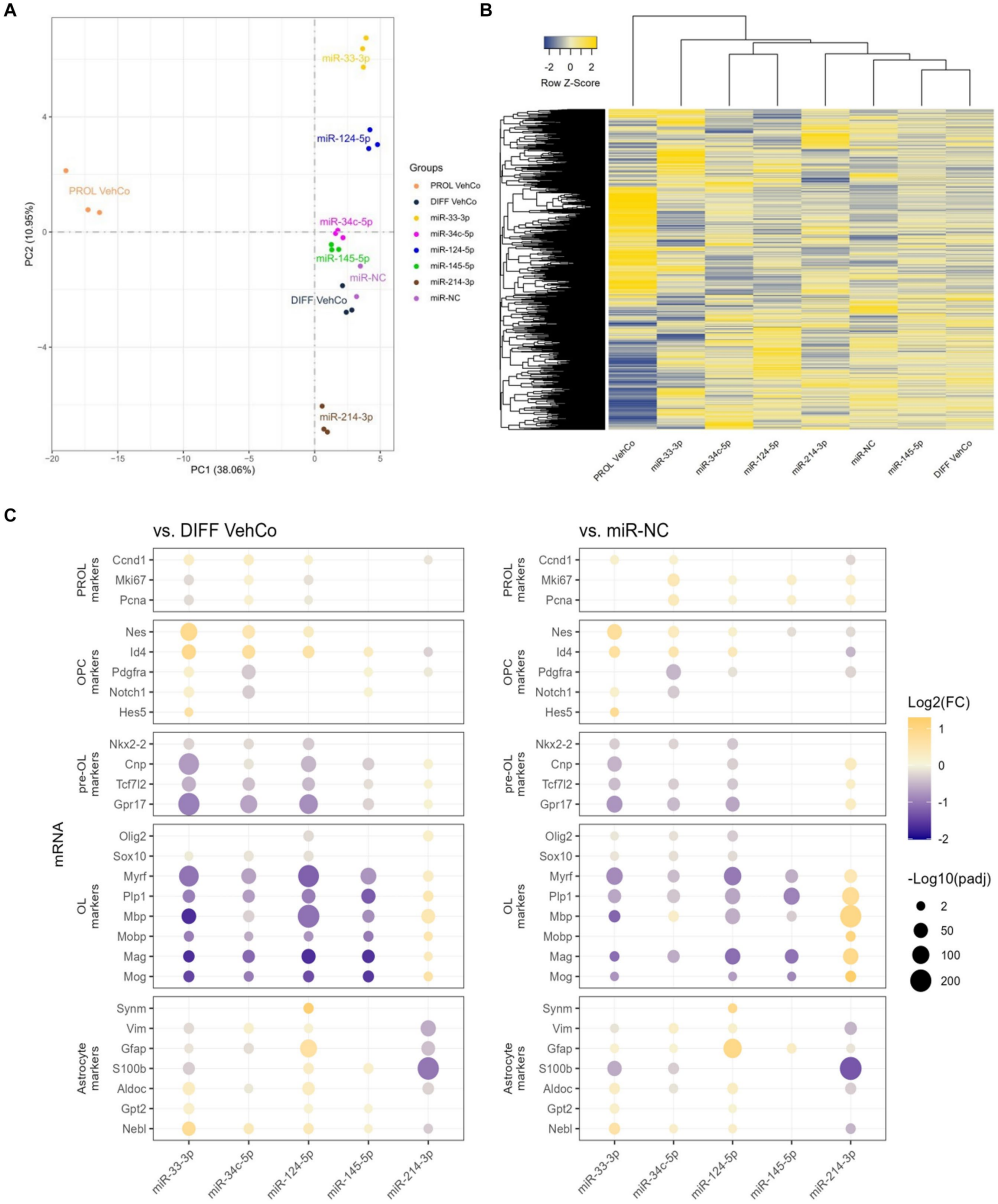
The effect of five microRNA mimics on the differentiation of CG-4 cells in RNA sequencing. **(A)** Principal component analysis was performed on the gene expression value [Fragments Per Kilobase Million (FPKM)] of all samples. In this plot, each sample is dispersed to the first and second principal components. The color of the dots corresponds to the microRNA species and controls, as indicated in the plot legend. **(B)** The heatmap shows the cluster analysis horizontally on the row-wise homogenized gene expression value (*z*-score of FPKM, represented by a blue-to-yellow color gradient from −2 to 2) and vertically on the different experimental conditions as labeled at the bottom of the heatmap. **(C)** This dot plot shows the fold change (FC) of the gene expression value of several proliferation (PROL), OPC, pre-OL, OL, and astrocyte markers, as labeled on the *y*-axis, to the differentiation vehicle control (DIFF VehCo) and the microRNA mimic negative control (miR-NC), resulting from the single transfection of each microRNA mimic, as labeled on the *x*-axis, in CG-4 cells in differentiation culture conditions. The color of each dot represents the fold change (expressed in logarithm base 2 [Log2(FC)]) of the relative expression, scaled by a blue-to-yellow color gradient from −2 to 1.3 (blue corresponding to a negative fold change, yellow to a positive), and its size represents the adjusted *p*-value (*p*_adj_) (expressed as minus logarithm base 10 [−Log10(*p*_adj_)]) as indicated in the plot legend. Only the significant data are depicted; thus, missing dots correspond to non-significant data. −Log10(*p*_adj_) ≥1.3 corresponds to the significance level of *p* ≤ 0.05. −Log10(*p*_adj_) ranges up to approximately 10–220 for certain targets. Data were from one experiment with each condition in triplicate, except miR-NC in duplicate. PROL VehCo, proliferation vehicle control; DIFF VehCo, differentiation vehicle control; PROL, proliferation; OPC, oligodendrocyte progenitor cells; pre-OL, premyelinating oligodendrocytes; OL, oligodendrocytes.

RNA sequencing was performed on all conditions in triplicates. One miR-NC replicate was excluded from the final analysis as it appeared to be an outlier masking the effects of the other conditions (see Methods section). Principal component analysis (PCA), performed on the gene expression value [Fragments Per Kilobase Million (FPKM)] of all samples, segregated well the different conditions, especially miR-33-3p, miR-124-5p, and miR-214-3p from the differentiation vehicle control and miR-NC conditions, and the proliferation vehicle control was distinctly segregated from all other conditions (Figure 6A). The first and second principal components covered only 38.06% and 10.95%, respectively, of the conditions’ variance. The gene expression, illustrated by the heatmap, was partially different between the microRNAs and the controls (Figure 6B). In accordance with the PCA, miR-145-5p clustered the closest to the differentiation vehicle control and miR-33-3p the furthest.

The significant dysregulation against the differentiation vehicle control and miR-NC, induced by each microRNA on genes involved in the different stages of OPC-to-OL differentiation is illustrated in Figure 6C (Mitew et al., 2014). These differentially expressed genes (DEG) evidenced the upregulation of OPC markers nestin (*Nes*) and/or *Id4* and the downregulation of premyelinating OL (pre-OL) markers NK2 homeobox 2 (*Nkx2-2*), *Tcf7l2*, *Cnp*, and G protein-coupled receptor 17 (*Gpr17*) and of mature OL markers [*Plp1*, *Myrf*, *Mbp*, *Mobp*, myelin-associated glycoprotein (*Mag*), myelin oligodendrocyte glycoprotein (*Mog*)] by miR-33-3p, miR-34c-5p miR-124-5p, and miR-145-5p, although the downregulation of the pre-OL markers by miR-145-5p was less pronounced, especially against miR-NC (Figure 6C). The opposite dysregulation of these markers was driven by miR-214-3p. Interestingly, miR-33-3p and miR-124-5p induced the expression of several astrocyte markers, such as aldolase fructose-bisphosphate C (*Aldoc*), glutamic-pyruvic transaminase 2 (*Gpt2*), and nebulette (*Nebl*). Immature astrocyte markers, vimentin (*Vim*) and *Gfap*, were upregulated by miR-124-5p but downregulated by miR-33-3p (Molofsky and Deneen, 2015; Jurga et al., 2021). All, except *Gpt2*, were downregulated by miR-214-3p as well (Figure 6C). Finally, all transfected cells, except miR-33-3p, had an increased proliferation rate [evidenced by the marker of proliferation Ki67 (*Mki67*), and *Pcna*] against miR-NC in RNA sequencing, while these markers were downregulated in miR-33-3p- and miR-124-5p-transfected cells against the differentiation vehicle control (Figure 6C). Nonetheless, Pcna expression did not significantly change among conditions at the protein level in immunocytochemistry (Figure 3A). The significantly increased expression of cyclin D1 (*Ccnd1*), *Mki67*, and *Pcna* against both controls further suggests active cell cycling in miR-34c-5p-transfected cells (Figure 6C).

**Figure 7:**
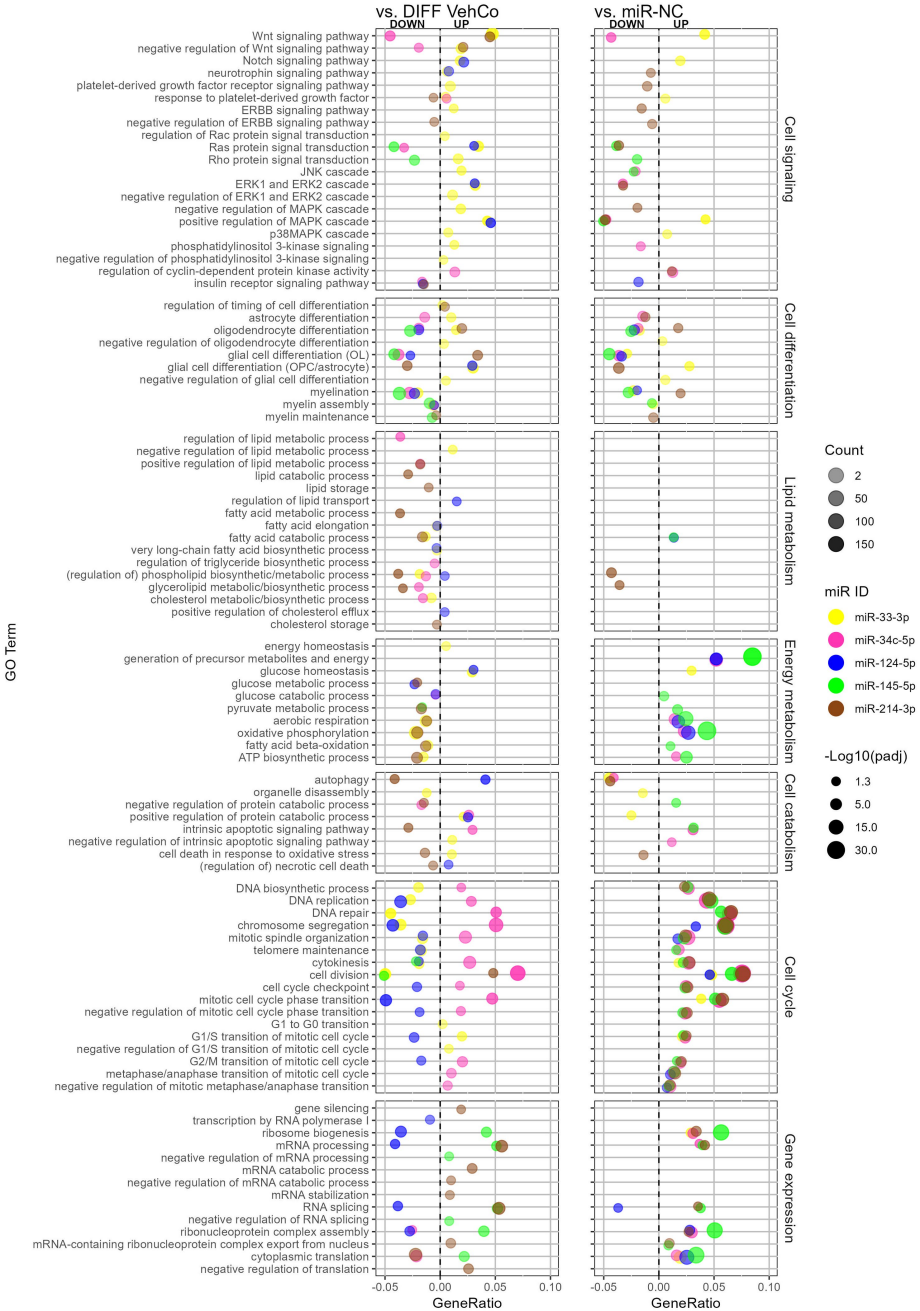
The effect of five microRNA mimics on the differentiation of CG-4 cells in RNA sequencing (Gene Ontology enrichment analysis). This dot plot shows the significant down- and upregulated Gene Ontology (GO) terms within the biological process for each microRNA to the differentiation vehicle control (DIFF VehCo) and the microRNA mimic negative control (miR-NC). The GO terms have been grouped by classes of biological processes as labeled on the right side of the plot. The gene ratio, which is the number of genes annotated to the GO term divided by the total number of down-/upregulated genes in the condition, is depicted on the *x*-axis, positively for upregulated GO terms, negatively for downregulated GO terms, separated by the vertical dotted line. The color of the dots corresponds to the microRNA species, the transparency to the absolute gene count, and the size to the adjusted *p*-value (expressed as minus logarithm base 10 [−Log10(*p*_adj_)]) as indicated in the plot legend. Data were from one experiment with each condition in triplicate, except miR-NC in duplicate.

We comprehensively compared the gene enrichment analysis of each microRNA against both the differentiation vehicle control and miR-NC conditions within the Gene Ontology (GO)/Biological Process (Figure 7). miR-214-3p supported OPC differentiation and myelination, whereas miR-33-3p, miR-34c-5p, miR-124-5p, and miR-145-5p countered these processes. The GO term “oligodendrocyte differentiation” was, however, upregulated by miR-33-3p against the differentiation vehicle control, although genes annotated to this GO term support OPC rather than OL physiology, such as *Notch1*, Hes family BHLH transcription factor (*Hes*) *1*, *Hes5*, *Id2*, *Id4*, and Leucine-rich repeat and immunoglobulin domain containing 1 (*Lingo1*). Note that the GO term “myelination” was not significantly dysregulated by miR-214-3p against the differentiation vehicle control, neither by miR-34c-5p against miR-NC. Additionally, astrocyte differentiation and glial cell differentiation with annotated genes rather linked to astrocytic physiology were upregulated by miR-33-3p and/or miR-124-5p but downregulated by miR-34c-5p and miR-214-3p. miR-33-3p upregulated signaling pathways involved in OPC/OL physiology, either in its proliferation (i.e., Wnt, Notch signaling pathway) or in both its proliferation and differentiation (i.e., mitogen-activated protein kinase (MAPK) and p38MAPK cascade) (Chew et al., 2010). It also upregulated the platelet-derived growth factor (Pdgfr) (proliferation) and extracellular signal-regulated kinase (ERK) and phosphoinositide-3-kinase (PI3K) (proliferation/differentiation) signaling pathways (Baron et al., 2000; Gaesser and Fyffe-Maricich, 2016), as well as the negative regulation of these signaling pathways, when compared to the differentiation vehicle control only. The other microRNAs affected fewer signaling pathways. miR-34c-5p downregulated the Wnt signaling pathway, while miR-124-5p induced the Notch signaling pathway and ERK cascade. miR-145-5p downregulated Ras protein signal transduction and miR-214-3p downregulated the “negative regulation of ERBB signaling pathway” (epidermal growth factor receptor (Egfr) tyrosine kinase family), both involved in differentiation.

Lipid metabolism was barely affected against miR-NC, except for miR-214-3p, which downregulated phospholipid/glycerolipid metabolism against both controls. Conversely, as compared to the differentiation vehicle control only, lipid metabolism was curtailed by miR-33-3p at the level of fatty acid, phospholipid, and cholesterol biosynthesis, by miR-34c-5p at the level of triglyceride/glycerolipid biosynthesis and phospholipid/cholesterol metabolism, and by miR-214-3p at the level of lipid/fatty acid catabolism and lipid/cholesterol storage, while miR-124-5p supported lipid/sterol transport.

Energy metabolism was negatively affected by miR-33-3p and miR-214-3p against the differentiation vehicle control but supported by miR-34c-5p, miR-124-5p, and miR-145-5p against miR-NC, as seen by the dysregulation of oxidative phosphorylation, aerobic respiration, ATP biosynthesis, and the generation of precursor metabolites. The energy homeostasis appeared, however, still safeguarded with miR-33-3p. miR-214-3p reduced autophagy and cell death in response to oxidative stress as well, while GO terms within autophagy and apoptosis were less consistent against both controls for the other microRNAs.

Cell cycle processes (cell division and cell cycle transition) were upregulated by all microRNAs against miR-NC but only by miR-34c-5p against the differentiation vehicle control and downregulated by miR-124-5p. Remarkably, the cyclin-dependent protein kinase activity was upregulated by miR-34c-5p against both controls. miR-33-3p impeded DNA biosynthesis, replication, repair, and telomere maintenance and supported the cell cycle transition only up to the G1/S phase compared to the differentiation vehicle control. Concerning gene expression, miR-145-5p and miR-214-3p supported (m)RNA processing and splicing against both controls, while miR-124-5p downregulated these against the differentiation vehicle control only. Furthermore, miR-145-5p supported the negative regulation of (m)RNA processing and splicing, while miR-214-3p negatively regulated translation and increased gene silencing against the differentiation vehicle control.

In conclusion, the RNA sequencing data supported our results obtained by RT-qPCR on independent experiments, phenotyping miR-33-3p, miR-34c-5p, and miR-124-5p transfected cells as late OPCs while miR-33-3p and miR-124-5p induced to a certain extent some astrocytic features as well. miR-145-5p transfected cells shifted closer to a pre-OL stage. All of them inhibited OPC differentiation and myelination, which was also supported by the decreased metabolism of various lipid species by both miR-33-3p and miR-34c-5p. miR-33-3p restrained DNA biosynthesis and energy metabolism, and miR-34c-5p sustained cell division, while miR-124-5p prevented cell cycle and gene expression compared to the differentiation vehicle control. GO enrichment analysis was overall poorer for miR-145-5p, but it downregulated Ras protein signal transduction. On the other hand, miR-214-3p fostered the differentiation of OPCs into a mature OL phenotype, which could be attributed to its regulation of Erbb signaling despite its negative impact on energy and lipid metabolism.

### 3.5 Several predicted mRNA targets possibly support the effect of each microRNA on OPC differentiation

We identified several potential mRNA targets of each microRNA using *in silico* analysis, at best predicted by three to four databases for both rat and human. We then verified whether RNA sequencing confirmed their downregulation (against the differentiation vehicle control and miR-NC) and whether they were correspondingly involved in OPC differentiation via a Pubmed search or their annotation to downregulated GO terms of interest (Supplementary Tables S2A,B). Their dysregulation by the other microRNAs was variable (data not shown).

**Figure 8:**
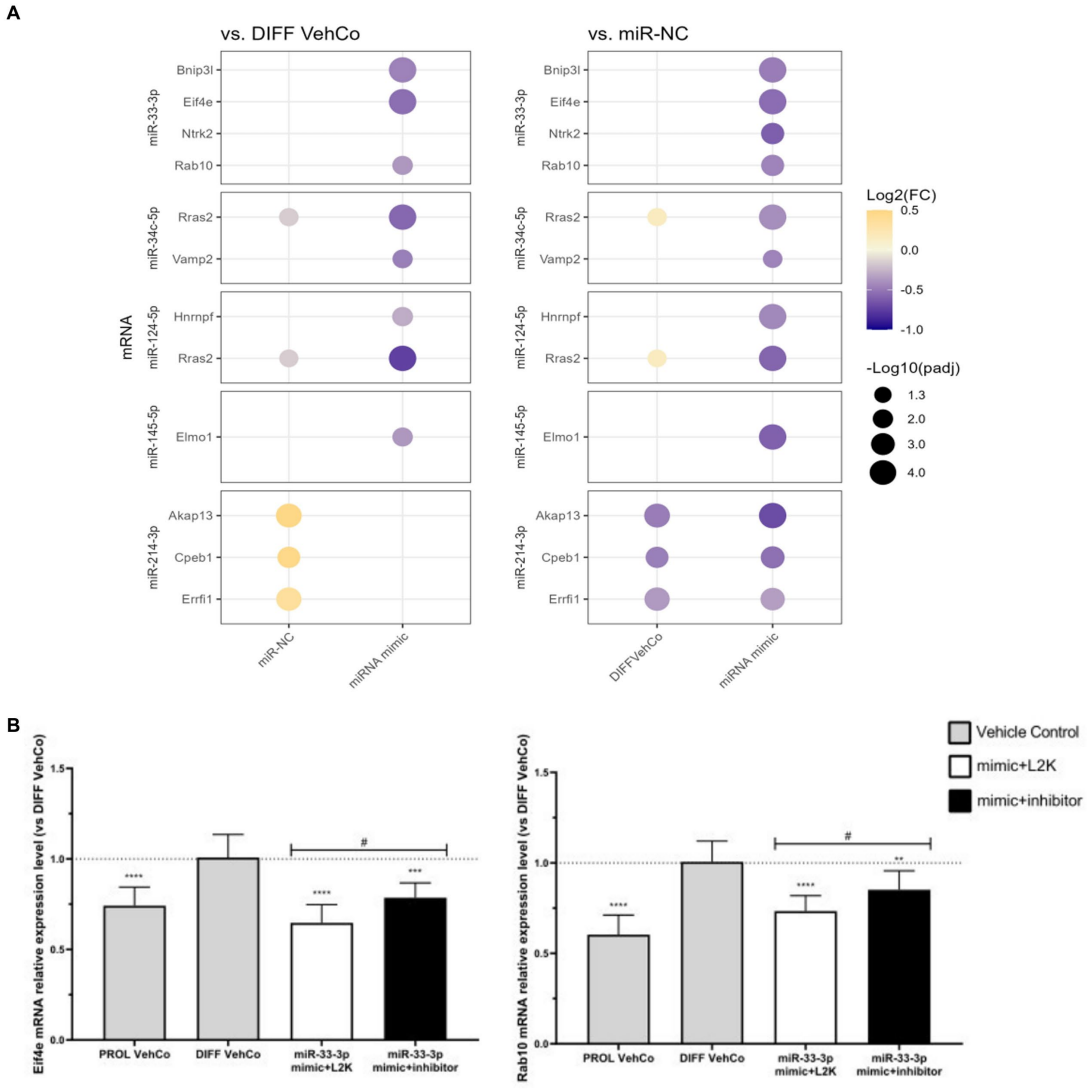
Potential mRNA targets of each microRNA verified in RT-qPCR. **(A)** This dot plot shows the fold change (FC) of the relative expression of potential mRNA targets of each microRNA as labeled on the *y*-axis, to the differentiation vehicle control (DIFF VehCo) and the microRNA mimic negative control (miR-NC), resulting from the single transfection of each microRNA mimic in CG-4 cells in differentiation culture conditions. The color of each dot represents the fold change (expressed in logarithm base 2 [Log2(FC)]) of the relative expression, scaled by a blue-to-yellow color gradient from −1 to 0.5 (blue corresponding to a negative fold change, yellow to a positive), and its size represents the adjusted *p*-value (*p*_adj_) (expressed as minus logarithm base 10 [−Log10(*p*_adj_)]) as indicated in the plot legend. The adjusted *p*-value was calculated by Dunn’s or Dunnett’s multiple comparison test to each control separately, i.e., the differentiation vehicle control and miR-NC, as recommended following a parametric/non-parametric one-way ANOVA on all conditions, including the proliferation vehicle control (not shown). Only the significant data are depicted; thus, missing dots correspond to non-significant data. −Log10(*p*_adj_) ≥1.3 corresponds to the significance level of *p* ≤ 0.05, −Log10(*p*_adj_) of 2.0 corresponds to *p* = 0.01, −Log10(*p*_adj_) of 3.0 to *p* = 0.001, and −Log10(*p*_adj_) of 4.0 to *p* = 0.0001. Data were from three experiments with each condition in triplicate. The corresponding bar plots showing both the statistically significant and non-significant results can be found in Supplementary Figure S5A. **(B)** The plots show the relative expression (mean with SD of 2^−ΔΔCt^) of *Eif4e* and *Rab10* to the differentiation vehicle control (DIFF VehCo, gray bar), resulting from the sequential transfection of miR-33-3p mimic followed (black bar) or not (white bar) by its inhibitor (as labeled on the *x*-axis) in CG-4 cells in differentiation culture conditions, as well as the proliferation vehicle control (PROL VehCo, gray bar). The dotted line represents the mean relative expression of the differentiation vehicle control to which the relative expression (2^−ΔΔCt^) of each sample was calculated. The adjusted *p*-value was calculated by Dunnett’s multiple comparison test to the differentiation vehicle control (*) and by Sidak’s multiple comparison test between the microRNA mimic and its inhibitor (#), as recommended following a parametric one-way ANOVA on all conditions: */# for *p* ≤ 0.05, ** for *p* ≤ 0.01, *** for *p* ≤ 0.001, **** for *p* ≤ 0.0001. Data were from three experiments with each condition in triplicate. L2K, Lipofectamine^™^ 2000; SD, standard deviation.

Hereafter, we confirmed the downregulation of several targets by RT-qPCR on three independent experiments: Bcl2 interacting protein 3 like (*Bnip3l*), eukaryotic translation initiation factor 4E (*Eif4e*), *Rab10*, a member of the Ras oncogene family (miR-33-3p), vesicle-associated membrane protein 2 (*Vamp2*) (miR-34c-5p), Ras-related 2 (*Rras2*) (miR-34c-5p and miR-124-5p), heterogeneous nuclear ribonucleoprotein F (*Hnrnpf*) (miR-124-5p) *Myrf*, and engulfment and cell motility 1 (*Elmo1*) (miR-145-5p) were significantly downregulated against both controls (Figure 8A; Supplementary Figure S5A). Note that *Vamp2* and *Rras2* were predicted *in silico* by three databases in rats but by only 2 or fewer in humans. For miR-34c-5p, *Rras2* was not significantly downregulated against miR-NC in RNA sequencing, but it was on independent RT-qPCR analysis. Furthermore, on independent RT-qPCR analysis, neurotrophic receptor tyrosine kinase 2 (*Ntrk2*) (miR-33-3p), A-kinase anchoring protein 13 (*Akap13*), cytoplasmic polyadenylation element binding protein 1 (*Cpeb1*), and Erbb receptor feedback inhibitor 1 (*Errfi1*) (miR-214-3p) were significantly downregulated against miR-NC only, but their expression was also significantly upregulated by miR-NC against the differentiation vehicle control, except for *Ntrk2*. Notably, *Akap13* showed a tendency toward downregulation against the differentiation vehicle control (Figure 8A; Supplementary Figure S5A). Finally, protein tyrosine phosphatase receptor type J (*Ptprj*) (miR-214-3p) was not significantly downregulated against neither of the controls in RT-qPCR, although it was in RNA sequencing (Supplementary Figure S5A).

The downregulation of these predicted targets was also corroborated by the sequential transfection of the microRNA mimic followed by a mock transfection, except for the miR-145-5p target *Elmo1* (Supplementary Figure S5B). However, only the expression of *Eif4e* and *Rab10* was significantly antagonized by the microRNA inhibitor compared to the miR-33-3p mimic (Figure 8B). For all the other investigated predicted targets, the microRNA inhibitor slightly, but non-significantly, tended to reverse or did not change the target’s expression level against its corresponding mimic (Supplementary Figure S5B).

Regarding miR-34c-5p, Notch receptor 1 (*Notch1*) and platelet-derived growth factor receptor A (*Pdgfra*) were targets predicted by both strategies. However, this was not in favor of the phenotype observed in miR-34c-5p-transfected CG-4 cells, as both Notch1 and Pdgfra support OPC proliferation and are expected to decrease during differentiation. Their downregulation by miR-34c-5p was verified by RNA sequencing and RT-qPCR analysis on three independent experiments (Figure 6C; Supplementary Figure S5A).

## 4 Discussion

We investigated the effect of 13 microRNAs previously identified as dysregulated in relapsing and/or remitting MS (Perdaens et al., 2020) on the differentiation of an OPC cell line called CG-4. Most of the screened microRNAs decreased OPC differentiation, while Dicer ablation in mice, which impedes microRNA maturation, markedly affects myelination, thereby suggesting that microRNAs would rather promote differentiation by inhibiting OPC proliferation (Dugas et al., 2010; Zhao et al., 2010; Emery and Lu, 2015). miR-146a-5p and miR-155-5p, for instance, two widely acknowledged microRNAs in MS, downregulated *Mbp* expression in our experimental setting. Unexpectedly, both the administration of exogenous miR-146a-5p as well as its knockout in mice improved cuprizone demyelination and/or EAE by promoting an anti-inflammatory phenotype of microglia/macrophages or reducing microglial/macrophagic infiltration in the corpus callosum, respectively, as well as increasing OPC differentiation and myelination (Zhang J. et al., 2017; Martin et al., 2018; Zhang et al., 2022). miR-155-5p, on the other hand, is upregulated during cuprizone demyelination, while treatment with its antagomiR improves it (Han et al., 2020; Koduru et al., 2023).

**Figure 9:**
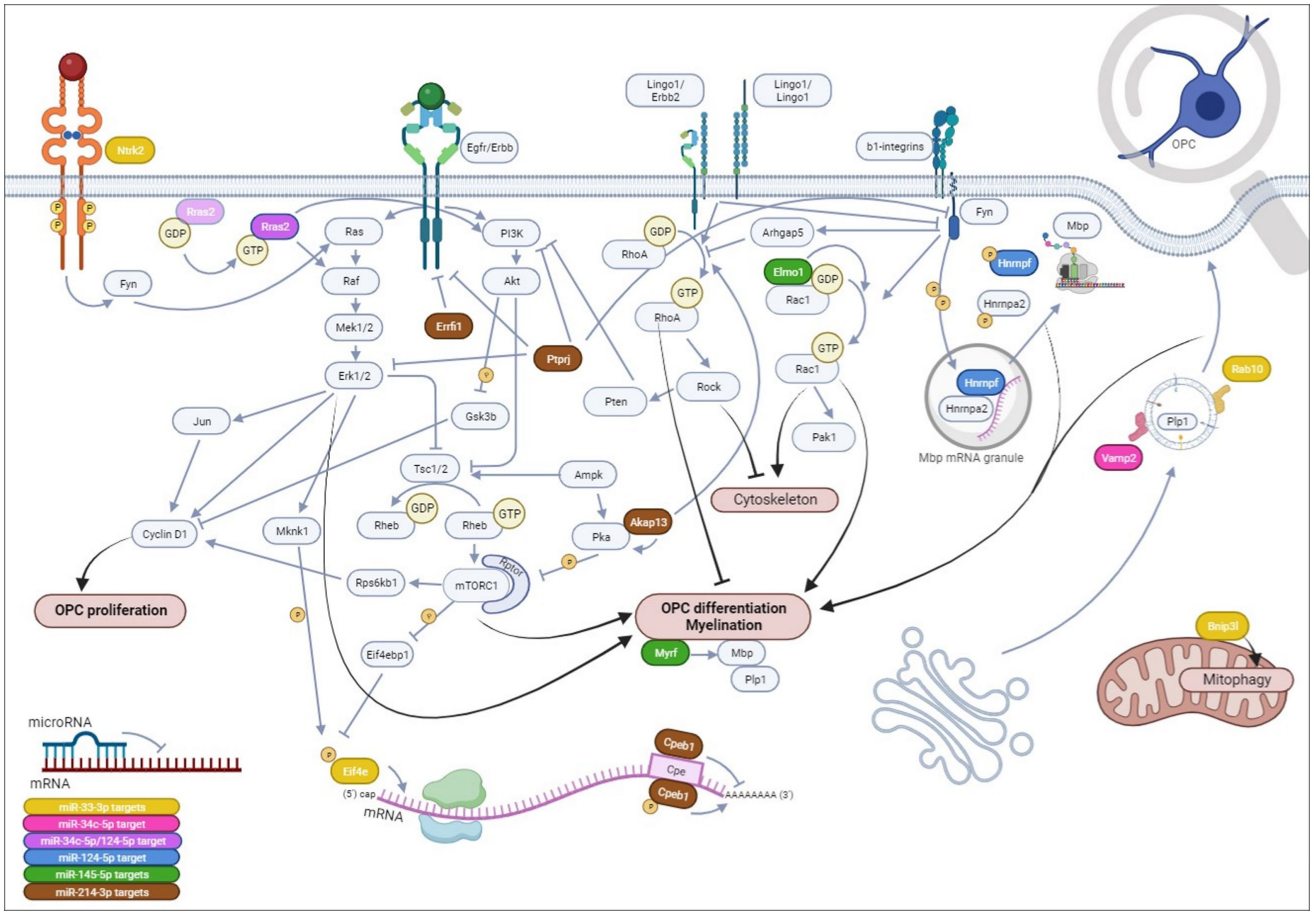
Hypotheses on the mechanistical/functional involvement of each microRNA and its potential mRNA targets on OPC differentiation. We have evidenced that miR-33-3p, miR-34c-5p, miR-124-5p, and miR-145-5p impede OPC differentiation, while miR-214-3p promotes it in CG-4 cells, an OPC cell line. Herein, we hypothesize that miR-33-3p (yellow) targets: (1) *Eif4e*, a translation initiation factor, part of the Eif4f complex, that once phosphorylated recognizes the mRNA 5′-terminal 7-methylguanosine (m7G) cap on OPC differentiation and myelin genes downstream PI3K/mTOR and ERK/MAPK signaling; (2) *Rab10*, a small GTPase involved in intracellular vesicle trafficking allowing plasma membrane elongation important for myelin ensheathment; (3) *Bnip3l* regulating mitochondrial function by mitophagy; and (4) more hypothetically *Ntrk2* upstream of the Ras/ERK/MAPK cascade. miR-34c-5p possibly targets *Vamp2* (pink), involved in intracellular vesicle targeting to the plasma membrane, leading to its expansion, and potentially *Rras2* (purple), a small GTPase involved in OPC differentiation by inducing ERK/MAPK and PI3K/Akt/mTOR signaling pathways. *Rras2* is also a predicted target of miR-124-5p. miR-124-5p is further predicted to target *Hnrnpf* (blue) that, upon phosphorylation by Fyn tyrosine kinase within *Mbp* transport granules, releases *Mbp* mRNA, allowing its translation within the cellular processes and its local interaction with the cytoplasmic membrane leaflets inducing myelin compaction. miR-145-5p (green) has already been proven to target *Myrf*, a key transcription factor of myelin genes, and possibly targets *Elmo1* as well, an activator of Rac1, a small GTPase (which is itself induced by Fyn tyrosine kinase by integrin engagement) inducing morphological OPC differentiation by acting on actin organization, or possibly via p21 activated kinase 1 (Pak1). Finally, miR-214-3p (brown), the only microRNA of our study inducing OPC differentiation, hypothetically targets (1) *Akap13*, a scaffolder of protein kinase A (Pka) that inhibits mTORC1 and an inducer of RhoA (which is itself inhibited by Fyn but induced by Lingo1) that was found to inhibit OPC differentiation by favoring stress fiber formation and actin depolymerization, or potentially by inducing Pten, an inhibitor of PI3K/Akt/mTOR signaling; (2) *Cpeb1*, acting as a translational repressor until it is phosphorylated or degraded, with its consensus binding sequence on several OPC/OL-related genes; (3) *Errfi1*, an Erbb receptor feedback inhibitor, and (4) *Ptprj*, a protein tyrosine phosphatase, both inhibiting Erbb signaling that can be involved in OPC differentiation. Ptprj can dephosphorylate ERK/MAPK, PI3K, and Fyn tyrosine kinase as well. Notably, the Erbb receptor tyrosine kinase family, existing of 4 isoforms, is important in OPC/OL physiology. Upon dimerization of two receptors within the Erbb family, which will determine the downstream signaling of the receptor tyrosine kinase, the signal will mainly be transduced intracellularly to the PI3K/Akt/mTOR signaling pathway, inducing OPC differentiation and myelination, or to the ERK/MAPK cascade, enhancing myelin thickness, although both have also been described as playing a role in OPC proliferation upon Pdgf induction through cyclin D1 (*Ccnd1*). OPC,oligodendrocyte progenitor cells, OL, oligodendrocytes. All references supporting these hypotheses are cited within the article, except Rac1/Pak1 and Lingo1/Erbb2 interactions (Parrini et al., 2002; Mi et al., 2013; Brown et al., 2021). *Created in* *BioRender.com*.

We then focused on five microRNAs—miR-33-3p, miR-34c-5p, miR-124-5p, miR-145-5p, and miR-214-3p—that exhibited the strongest dysregulation of the differentiation markers. Interestingly, miR-33-3p, miR-34c-5p, and miR-124-5p have not been functionally investigated in the scope of multiple sclerosis so far. Only miR-214-3p promoted OPC differentiation into more mature OLs, while the other four microRNAs arrested it, each to a different extent, at a late progenitor (miR-33-3p, miR-34c-5p, and miR-124-5p)-premyelinating (miR-145-5p) stage. We further explored the phenotype of these CG-4 cells transfected with different microRNAs through the significant DEG and GO terms in RNA sequencing. We identified several potential targets by *in silico* analysis that were also downregulated in RNA sequencing (DEG and/or GO terms) and confirmed some by RT-qPCR analyses on independent experiments. Based on our data, we finally propose mechanistic hypotheses for the observed effects on OPC differentiation of each microRNA via the predicted mRNA targets, some of which remain so far unexplored in OPC/OL physiology (Figure 9).

### 4.1 miR-33-3p strongly inhibits OPC differentiation, possibly by altering PI3K/mTOR signaling, membrane trafficking, and/or mitochondrial function

miR-33-3p unveiled the strongest inhibitory effect on OPC differentiation as seen by the downregulation of *Mbp* and *Plp1*, although “oligodendrocyte differentiation” was upregulated against the differentiation vehicle control in GO enrichment analysis but downregulated against miR-NC, whereas its negative regulation was commonly upregulated. In fact, OPC-related genes, such as *Notch1*, *Hes1*, *Hes5*, *Id4*, and *Lingo1*, were annotated to these GO terms (Mitew et al., 2014). Furthermore, myelination was downregulated in GO against both controls. Herein, miR-33-3p potentially targets *Rab10* and *Eif4e*, as supported by *in silico* analysis and by their significant downregulation in RNA sequencing and independent RT-qPCR analyses. Moreover, they were the only predicted targets significantly antagonized by the microRNA inhibitors. They could thus directly affect OPC differentiation and/or myelination. Rab10, a small GTPase of the Rab family, is involved in the addition of proteins and lipids to the plasma membrane by regulating intracellular vesicle trafficking necessary for OL maturation and myelin sheath formation (Hutagalung and Novick, 2011; Zhang Z. et al., 2017). Its inactivation has been involved in neurodegeneration due to lysosomal dysfunction in Parkinson’s disease (Di Maio et al., 2018). On the contrary, in the context of Alzheimer’s disease, the knockdown of *RAB10* is protective by decreasing amyloid beta 42 levels (Ridge et al., 2017). Translation initiation factor Eif4e is released downstream of PI3K/mammalian target of rapamycin (mTOR) signaling by phosphorylation of its repressor, binding protein Eif4ebp1, conceivably increasing the translation of myelin proteins (Bibollet-Bahena and Almazan, 2009; Coelho et al., 2009). Furthermore, Ntrk2 signaling promotes via the Ras/ERK cascade OPC differentiation and remyelination in the demyelinating cuprizone mice model (Peckham et al., 2016; Fletcher et al., 2018; Huang et al., 2020). *Ntrk2* downregulation was, however, only confirmed against miR-NC by independent RT-qPCR analysis. miR-33-3p has not been studied in OPC differentiation so far, and the literature on miR-33-3p is sparse, possibly because it is the minor product of the mir-33 precursor. Conversely, it arrests the proliferation of rat pheochromocytoma PC12 cells in favor of their differentiation toward choline acetyltransferase-positive neuron-like cells (Shan et al., 2021).

Wnt and Notch signaling, supporting OPCs, were the only pathways upregulated against both differentiation vehicle control and miR-NC (Wang et al., 1998; Ortega et al., 2013). Of note, two signaling pathways linked to both OPC proliferation (downstream Pdgfra) and differentiation, namely the ERK/MAPK cascade and PI3K signaling pathway, as well as their negative regulation, were upregulated against the differentiation vehicle control only (Baron et al., 2000; Gaesser and Fyffe-Maricich, 2016). Accordingly, a higher proportion of miR-33-3p-transfected cells seemed A2B5^+^, although this was not statistically significant. Cell division was downregulated in GO due to reduced DNA biosynthesis/repair and telomere maintenance.

GO indicated that the biosynthesis of very long-chain fatty acids, acyl-CoA, phospholipids, and cholesterol, which are structural lipids/elements of myelin (Poitelon et al., 2020) were downregulated in miR-33-3p-transfected cells. Herein, fatty acyl-CoA reductase 1 (*Far1*), a predicted target of miR-33-3p we did not further investigate, is involved in glycerophospholipid (plasmalogen) synthesis (Honsho et al., 2010). Moreover, miR-33-5p, the more abundant product, reduces cholesterol efflux and fatty acid degradation (Gerin et al., 2010). Interestingly, the mir-33 precursor is located in an intron of the sterol regulatory element-binding protein 2 (*SREBP2*) locus, a sterol-sensing transcription factor (Gerin et al., 2010).

Energy metabolism is important in the myelination process (Tepavčević, 2021). Although energy homeostasis, especially through glucose homeostasis, appeared safeguarded in miR-33-3p-transfected cells, the generation of precursor metabolites was downregulated, paralleling the downregulation of oxidative phosphorylation and aerobic respiration against the differentiation vehicle control, resulting in ATP biosynthesis deficiency. Additionally, we confirmed the downregulation of *Bnip3l*, another predicted target of miR-33-3p, that is upregulated in differentiating OPCs and induces mitophagy to allow proper mitochondrial function (Itoh et al., 2003; Yazdankhah et al., 2021). Interestingly, *Bnip3l* was also annotated to downregulated GO terms as “organelle disassembly” (against both controls) and “autophagy” (against miR-NC).

Finally, miR-33-3p-transfected CG-4 cells arrested for OPC differentiation acquired astrocytic features as supported by the upregulation of GO term “astrocyte differentiation” and of several astrocytic markers (*Aldoc*, *Gpt2*, and *Nebl*) in RNA sequencing, while immature astrocytic markers *Vim* and *Gfap* were downregulated. Herein, miR-33-3p is predicted to target nude neurodevelopment protein 1 like 1 (*Ndel1*), which is critical for neuronal migration but was also found to inhibit astrocyte differentiation, although to a much lesser extent than its ohnologue, Nde1 (Pei et al., 2014).

### 4.2 miR-34c-5p sustains cell division, although it targets *Pdgfra* and *Notch1* and may affect signal transduction in differentiation pathways as well as membrane expansion

miR-34c-5p downregulated the differentiation markers *Mbp* and *Plp1*, albeit to a lesser extent than miR-33-3p. This was supported by the downregulated GO term “oligodendrocyte differentiation.”

We verified the downregulation of miR-34c-5p target *Vamp2* by RNA sequencing and independent RT-qPCR analyses. Vamp2, a soluble N-ethylmaleimide sensitive factor attachment protein receptor (SNARE) protein, is involved in exocytosis by guiding the fusion of the vesicular and plasma membranes. Herein, Vamp2/3 was necessary for membrane expansion but not for OPC differentiation, even though it was experimentally inactivated in premyelinating OLs through *Cnp*-dependency (Lam et al., 2022). Conversely, in another study, Vamp2/3-cleavage arrested OPC at a premyelinating, early stage (Fekete et al., 2023). This was also noted in our study, as RNA sequencing showed that *Cnp* was already downregulated in miR-34c-5p-transfected CG-4 cells, although this was not confirmed by independent RT-qPCR analyses. Hence, its involvement in OPC differentiation might warrant further research. The interaction of miR-34c-5p and *Vamp2* has previously been validated by luciferase assay, and Vamp2 was found to be significantly less expressed at the protein level in the brain of Alzheimer patients and in amyloid beta-42-treated rat hippocampal neurons (Pham et al., 2010; Hu et al., 2015). Overexpression of miR-34c-5p impairs dendritic length, branching morphology, and synaptic function in neurons (Hu et al., 2015; Kao et al., 2018). On the contrary, it has also been positively linked to neurogenesis, and it is neuroprotective by reducing mitochondrial dysfunction, oxidative stress, and neuronal apoptosis (Miñones-Moyano et al., 2011; Jauhari et al., 2018; Tu and Hu, 2021).

GO enrichment analysis was further mainly marked by the upregulation of GO terms related to the cell cycle (cell division, cell cycle phase transition, DNA replication, and chromosome segregation). Accordingly, miR-34c-5p-transfected cells tended to be more A2B5^+^ and the cell cycle markers, *Mki67* and *Pcna*, were significantly upregulated in RNA sequencing. Herein, the cyclin-dependent protein kinase activity [e.g., cyclin D1 (*Ccnd1*), a cell cycle coordinator] was upregulated against both differentiation vehicle control and miR-NC (Nobs et al., 2014). Cyclin D1 is induced by several pathways, namely Wnt/beta-catenin, ERK-MAPK/Jun (Jun proto-oncogene, AP-1 transcription factor subunit), PI3K/Akt/Gsk3b (glycogen synthase kinase 3 beta), and PI3K/Akt/mTOR, which can all be induced by Pdgf in OPCs (Baron et al., 2000; Chew et al., 2010, 2011; Choi and Anders, 2014). Moreover, Wnt, Pdgfra, and Notch1 signaling support OPC proliferation and their downregulation accompanies OPC differentiation (Noble et al., 1988; Wang et al., 1998; Ortega et al., 2013). However, in our study, the Wnt signaling pathway was downregulated by miR-34c-5p in GO, and both *Notch1* and *Pdgfra*, proven mRNA targets of miR-34c-5p (Bai et al., 2017; Wei et al., 2021), were significantly downregulated in RNA sequencing and RT-qPCR analyses on independent experiments. Of note, *Hes5*, the downstream transcription factor of Notch1 was not differentially expressed, and *Nkx2-2* that induces early differentiation by directly repressing *Pdgfra* (Zhu et al., 2014) was downregulated as well in our experimental condition (Figure 6C). It is, therefore, not clear which pathway supports their proliferation. This should be further explored as, for example, Jun N-terminal kinase (Jnk) interacting protein 1 [also called mitogen-activated protein kinase 8 interacting protein 1 (*Mapk8ip1*)] and *Jun*, elements of the Jnk signaling pathway involved in OPC proliferation, were slightly but significantly upregulated, as well as *Ntrk3*, that has been linked to both OPC proliferation/survival and differentiation (data not shown) (Kumar et al., 1998; Chew et al., 2010; Baldassarro et al., 2023). However, miR-34c-5p was also identified as anti-proliferative in glioblastoma and osteosarcoma cells (Wu et al., 2013; Wang Y. et al., 2019).

Hence, to potentially explain that miR-34c-5p-transfected cells do not engage in differentiation, we demonstrated the downregulation of *Rras2* mRNA, a small GTPase of the Ras-related subfamily, that supports OPC differentiation through the PI3K/Akt/mTOR and ERK1/2-MAPK signaling pathways. For both pathways, the GO terms were downregulated compared to miR-NC. *Rras2* knockout results in an increase in immature OLs (Sanz-Rodriguez et al., 2018; Alcover-Sanchez et al., 2020).

Finally, the metabolism of specific lipid species, i.e., phospholipids, glycerolipids, and cholesterol, was downregulated as well, compared to the differentiation vehicle control only.

### 4.3 miR-124-5p possibly impedes signal transduction in differentiation pathways as well as *Mbp* mRNA granule transport

miR-124-5p caused the second strongest downregulation of differentiation markers *Mbp* and *Plp1*, supported by the downregulation of the GO term “oligodendrocyte differentiation.” In miR-124-5p-transfected cells, the insulin receptor signaling pathway was downregulated against both differentiation vehicle control and miR-NC, while Notch and Ntrk signaling were upregulated as compared to the former only. Insulin-like growth factor 1 is seemingly involved at all stages of OPC development, proliferation, survival, and differentiation (Zeger et al., 2007). All cell cycle processes were also reduced as compared to the differentiation vehicle control. Lipid biosynthesis was not affected, and energy metabolism was overall positively balanced.

Little is known about miR-124-5p, while increased neuronal levels of miR-124-3p, the major product of mir-124 precursor, accompanied hippocampal demyelination in MS brains (Dutta et al., 2013). miR-124-3p also promotes neurogenesis, and its downregulation has been linked to Alzheimer’s, Parkinson’s, and Huntington’s disease pathogenesis (Lim et al., 2005; Cheng et al., 2009; Zhang W.-H. et al., 2023). However, its upregulation in motor neurons could also cause early apoptosis (Vaz et al., 2021).

We confirmed the downregulation of *Hnrnpf*, a predicted target of miR-124-5p, by RNA sequencing and RT-qPCR analyses on independent experiments. Hnrnpf, alongside Hnrnpa2, binds *Mbp* mRNA in RNA transport granules and silences its translation along transport to the cell periphery, where the phosphorylation of Hnrnpf by Fyn proto-oncogene 1 Src family tyrosine kinase (Fyn) releases *Mbp* mRNA allowing its on-site translation in the cellular processes whereafter it can easily interact with the apposing cytoplasmic leaflets for myelin compaction (White et al., 2012; Aggarwal et al., 2013). Hnrnpab is similarly involved in the RNA transport of *Mbp*, and *Mbp* mRNA was localized in the cell body instead of cellular processes by its silencing (Raju et al., 2008). Interestingly, RNA sequencing in our study showed a bigger negative fold change for *Hnrnpab*; however, while it met our selection criteria as a predicted target of miR-124-5p in rats, it was only predicted by two databases in humans.

Similar to miR-34c-5p, *Rras2* is a predicted target of miR-124-5p but in rats only. We evidenced its downregulation by RNA sequencing and independent RT-qPCR analyses.

Finally, we evidenced the overexpression of *Gfap* by miR-124-5p at the mRNA level by independent RT-qPCR analyses and at the protein level by immunocytochemistry, which was further supported by the upregulation of both immature [synemin (*Synm*), *Vim*, and *Gfap*] and general/mature (*Aldoc*, *Gpt2*, and *Nebl*) astrocytic markers in RNA sequencing. miR-124-5p is predicted to target Lysine acetyltransferase 5 (*Kat5*) in rats, and Kat5-deficiency induces a neurogenesis-to-gliogenesis switch *in vivo* and an increase of Gfap^+^ astrocytes *in vitro* (Tominaga et al., 2023).

### 4.4 miR-145-5p targets a key transcription factor for OPC differentiation but may also indirectly affect signal transduction by a small GTPase

miR-145-5p inhibited the differentiation of CG-4 cells as well, as evidenced by the downregulation of *Mbp* and *Plp1* and the GO terms “oligodendrocyte differentiation” and “myelination.” We hereby confirm, using other techniques and in a different cell model, what was previously demonstrated (Kornfeld et al., 2021). Kornfeld, e.a., validated *Myrf* as a direct target of miR-145-5p on primary OPCs by exploring the effects of its microRNA inhibitor. We showed its downregulation in RNA sequencing and independent RT-qPCR analyses, although it was downregulated by the other differentiation-impeding microRNAs as well. Myrf is indispensable for OL development and myelin maintenance. Along with its inducer Sox10, it separately or cooperatively activates OL genes and blocks the expression of Sox10-dependent OPC genes (Bujalka et al., 2013; Hornig et al., 2013; Aprato et al., 2020).

Unexpectedly, miR-145-5p-transfected cells were clustered closest to the controls, the differentiation vehicle control, and miR-NC, as seen by the PCA and on the heatmap of the RNA sequencing. GO dysregulation was also limited. Ras and Rho protein signal transduction was downregulated against both controls. Herein, we demonstrated the downregulation of *Elmo1*, a predicted target of miR-145-5p and an activator of Rac family small GTPase 1 (Rac1) (Mikdache et al., 2020). Rac1 supports morphological OPC differentiation and is also involved in the organization of actin within the cytoskeleton (Liang et al., 2004).

Overall, lipid metabolism was not affected. Energy metabolism and cell cycle processes were upregulated against miR-NC only, while mRNA processing was upregulated against both controls. Conversely, the downregulation of miR-145-5p in glioblastomas supports tumor growth and tumor cell invasion (Lee et al., 2013; Rani et al., 2013).

### 4.5 miR-214-3p unexpectedly promotes the differentiation of CG-4 cells, a matter of timing?

In our study, we repeatedly (by single, double, and sequential transfection) demonstrated that miR-214-3p induced CG-4 cell differentiation, as evidenced by the upregulation of *Plp1*, *Mbp*, and *Mobp*. miR-214-3p has, however, long been postulated to hinder OPC differentiation as it is predicted to target *Mobp*, an important protein for myelin compaction and structure, although this has never been functionally investigated (Yamamoto et al., 1999; Letzen et al., 2010). Interestingly, miR-214-3p promoted the migration and myelination of Schwann cell-like cells derived from human amniotic mesenchymal stem cells by targeting *Jun* and could improve peripheral nerve regeneration (Chen et al., 2019). Furthermore, miR-214-3p promotes the differentiation of neural progenitor cells, while the opposite has been described as well. It is neuroprotective and reduces both neuronal apoptosis and autophagy via several mRNA targets (Zhang et al., 2016; Shu et al., 2017; Mellios et al., 2018; Wu et al., 2019; Zhang M. et al., 2023). Notably, its effect on neural progenitor cell proliferation and neuronal apoptosis was found to be mediated by targeting *Pten*, an inhibitor of PI3K/Akt signaling, and thus inducing this pathway, which is involved in both OPC proliferation and differentiation (Baron et al., 2000; Zhang et al., 2013; Gaesser and Fyffe-Maricich, 2016; Mellios et al., 2018; Wu et al., 2019). *Pten* was, however, not downregulated by miR-214-3p in CG-4 cells, according to our RNA sequencing data. miR-214-3p has also been characterized as a tumor suppressor in gliomas (Wang et al., 2014; Tang et al., 2015).

Understanding the changes in cell fate induced by miR-214-3p through GO enrichment analysis was further challenging. The “negative regulation of Erbb signaling” was downregulated against both controls, with *Errfi1* and *Ptprj* as genes annotated to this GO term. Erbb receptors are involved in OPC proliferation and differentiation, depending on their dimerization partner (Galvez-Contreras et al., 2013). Other pathways, such as Pdgfr signaling, Ras signal transduction, and ERK1/2 cascade, were downregulated against miR-NC only. The phospholipid and glycerolipid metabolism, as well as autophagy, were downregulated, but cell division was upregulated against both controls, while energy metabolism was largely and negatively affected as compared to the differentiation vehicle control only.

We hypothesized that miR-214-3p targets *Errfi1* and growth factor receptor bound protein 2 (*Grb2*), as predicted by *in silico* analysis and as seen by their downregulation in RNA sequencing. Errfi1 inhibits Egfr signaling, while Grb2 binds to Egfr and induces Erk/Jun signaling. A similar microRNA-mRNA target mechanism has been described for miR-200a, resulting in a shift of Egfr/Erk/Jun signaling toward Egfr/PI3K/Akt/mTOR signaling and enhanced OPC differentiation (Santra et al., 2016). Additionally, Ptprj dephosphorylates and inactivates receptor tyrosine kinases as Egfr and possibly other Erbb receptors, ERK1/2, PI3K, and Fyn tyrosine kinase as well (Berset et al., 2005; Tsuboi et al., 2008; Sacco et al., 2009; Aya-Bonilla et al., 2014; Schneble et al., 2017). Therefore, the downregulation of *Ptprj* may maintain the activity of these protein kinases and thus subsequently promote OPC differentiation through ERK/MAPK and/or PI3K/Akt/mTOR signaling and by facilitating *Mbp* translation in RNA transport granules (White et al., 2012; Galvez-Contreras et al., 2013; Gaesser and Fyffe-Maricich, 2016).

Alternately, Cpeb1 binds to a consensus sequence, called cytoplasmic polyadenylation element (CPE), within the 3’UTR of a mRNA to repress its translation until it is itself phosphorylated or degraded (Mendez and Richter, 2001). Cpeb1 contributes to dendritic branching and synaptic plasticity in neurons and induces the differentiation of glioma stem cells by binding to *Hes1* (Bestman and Cline, 2008; Yin et al., 2014). We have identified the CPE consensus sequence (5′-UUUUUAU-3′) within the 3’UTR of several mRNAs related to OPC proliferation (*Notch1*, *Hes1*, *Id2*, and *Id4*) and differentiation (*Olig1*, *Tcf7l2*, *Yy1* transcription factor, and *Mbp*), all of which were confirmed by the bioinformatic resource^5^ in human and/or mice (no data available for rats), except for *Yy1*. Therefore, if miR-214-3p targets *Cpeb1*, it would increase their translation. Finally, Akap13 inhibits mTORC1 by scaffolding protein kinase A and induces Ras homolog family member A (RhoA) activity, both of which impede OPC differentiation (Klussmann et al., 2001; Liang et al., 2004; Mi et al., 2005; Abdul Azeez et al., 2014; Gaesser and Fyffe-Maricich, 2016; Zhang et al., 2021). Interestingly, RhoA activation favors the formation and contraction of stress fibers and the depolymerization of actin via downstream Rho-associated coiled-coil containing protein kinase (Rock), thereby inhibiting process expansion and differentiation in OPCs (Pedraza et al., 2014). RhoA/Rock can also possibly inhibit PI3K/Akt through induction of Pten, although this has not yet been evidenced in OPCs (Yang and Kim, 2012; Tan et al., 2018; Wang et al., 2022). Remarkably, the roles of Akap13, Ptprj, and Cpeb1 in OPCs/OLs remain unexplored.

*Akap13*, *Cpeb1*, *Errfi1*, *Grb2*, and *Ptprj* emerge as strong candidates mechanistically supporting OPC differentiation induced by miR-214-3p. Although their downregulation was evidenced by RNA sequencing, we could not confirm it by RT-qPCR analyses on independent experiments, except for *Akap13*, *Cpeb1*, and *Errfi1* against miR-NC (data not shown for *Grb2*). Nonetheless, it is possible that the expression of a direct target remains unaffected at the mRNA level, as the microRNA might restrain its translation rather than cause its degradation (Bartel, 2009). Notably, only the expression of *Akap13* tended to be slightly decreased compared to the differentiation vehicle control. In fact, as it was not consistently downregulated in one of the three independent experiments, in which *Plp1* and *Mbp* were also slightly less, but still significantly, upregulated, possibly due to a later passage of the cells, we verified and confirmed by RT-qPCR its downregulation in the experiment sent for RNA sequencing (data not shown). Therefore, exploring the expression of these candidates and the pathways they are involved in at a protein level, as well as their direct interaction with miR-214-3p by a dual luciferase assay, would be of interest.

The induction of OPC differentiation might, however, be a matter of timing. The differentiation markers were more potently induced by miR-214-3p than miR-219a-5p in the short, single transfection protocol (1 + 2 days), while their upregulation by miR-214-3p was lower than by miR-219a-5p in the long, double transfection protocol (1.5 + 6 days). Therefore, miR-214-3p could induce initial OPC differentiation, but it might not be able to maintain it in the long term, as its effect seemed to slow down. Similarly, miR-138-5p was found to induce early phases of OPC differentiation but inhibit it at later stages (Dugas et al., 2010). This remains largely hypothetical, as both protocols are very different and should be further investigated with precise kinetics.

### 4.6 Does endogenous microRNA expression mirror the effect of the transfected microRNAs?

miR-33-3p was endogenously not differentially expressed in both basal proliferating and differentiating CG-4 cells (without any treatment other than the medium change), which may not directly imply its endogenous involvement in OPC differentiation.

Endogenous miR-34c-5p and miR-124-5p were downregulated in differentiating CG-4 cells, consistent with their inhibitory effect on OPC differentiation identified in our study. On the contrary, endogenous miR-145-5p was upregulated in differentiating CG-4 cells, while we showed that its transfection interfered with OPC differentiation. Finally, miR-214-3p was not detectable in proliferating nor differentiating CG-4 cells.

The expression of miR-33-3p and miR-124-5p has not been described in OPC differentiation/development so far, possibly because they are the minor product of their corresponding precursor microRNA. miR-219-5p, miR-145-5p, and miR-214-3p were found upregulated and miR-34c-5p slightly downregulated during OL maturation from primary rat A2B5^+^ OPCs to premyelinating Galc^+^ OLs (Lau et al., 2008). Conversely, during the maturation of human embryonic stem cells, miR-145-5p and miR-214-3p, even more strongly, were increased at the early O4^+^ OPC stage but already decreased from the mid-Galc^+^ OPC stage and slightly increased again in mature OLs (Letzen et al., 2010). Similarly, miR-145-5p decreased gradually within a few hours of differentiation induction in rat OPCs, but here, it remained low up to 5 days of differentiation (Kornfeld et al., 2021).

Thus, the endogenous downregulation of miR-34c-5p and miR-124-5p possibly mirrors their inhibition of OPC differentiation. On the contrary, the endogenous expression of miR-145-5p noticeably varies following a time and cell-stage-specific course, as evidenced by others (Lau et al., 2008; Letzen et al., 2010; Kornfeld et al., 2021). We acknowledge the possibility of missing its downregulation during CG-4 cell differentiation by capturing its expression at a single timepoint only. However, these microRNAs might also be expressed differently by OPCs/OLs or other cell types in physiological and/or pathophysiological conditions of the central nervous system. Determining their cellular source could therefore further inform on their physiological and pathophysiological role.

### 4.7 Does MS-related microRNA dysregulation mirror the effect of the transfected microRNAs?

In our previous study on microRNA dysregulation in the CSF, miR-33-3p and miR-214-3p were downregulated in remitting MS patients and miR-34c-5p and miR-124-5p in relapsing MS patients, while miR-145-5p was upregulated in relapsing patients (Perdaens et al., 2020). As both miR-33-3p and miR-145-5p inhibit OPC differentiation, their respective downregulation during remission and upregulation during relapse potentially support the pathogenesis of MS within the CSF. However, this is less clear considering the dysregulation of miR-34c-5p and miR-124-5p. On the contrary, the downregulation of miR-214-3p, a promoter of OPC differentiation, in the CSF of remitting MS patients could underpin remyelination defects. It might as well be a matter of compartment differences, as miR-214-3p was also found upregulated in active and even more in inactive white matter lesions of MS patients (Junker et al., 2009).

miR-33-3p and miR-124-5p have not been linked to MS so far, except in our previous study (Perdaens et al., 2020). An extended bioinformatic analysis linked miR-34c-5p, identified only by our previous study so far, to many MS-related genes, including *MAPK1*. This characterized it as a risk microRNA in MS (Su et al., 2022), possibly supporting its inhibitory effect on OPC differentiation, as demonstrated by our results. However, in the CSF, miR-34c-5p was downregulated during relapses. miR-145-5p, which was also found upregulated in plasma, serum, and peripheral blood mononuclear cells of MS patients (Keller et al., 2009; Søndergaard et al., 2013), has been characterized as a pro-inflammatory [by targeting immune response limiting factors, semaphorin 3A and cytotoxic T lymphocyte associated protein 4 (*CTLA4*)] and an anti-OPC differentiation (by targeting *Myrf*) microRNA, and is thus potentially supportive of MS pathogenesis (Fayyad-Kazan et al., 2012; Rezaeepoor et al., 2018; Kornfeld et al., 2021). Conversely, a bioinformatic-based network established the sponging of miR-145-5p by an upregulated long non-coding RNA, taurine upregulated 1 (*TUG1*), in the periplaque regions in MS brains, leading to the upregulation of catenin delta (*CTNND1*), a potential MBP-interacting protein (Smirnova et al., 2021; Sabaie et al., 2022).

Therefore, the data from our study and existing literature suggest a potential link between the dysregulation and a pathophysiological role of miR-33-3p and miR-145-5p in MS. Furthermore, the opposite dysregulation of miR-214-3p in the CSF (downregulated) and the white matter lesions (upregulated) could be related to repair mechanisms potentially supporting initial OPC differentiation (by its upregulation) or oppositely to their impairment resulting in remyelination defects (by its downregulation).

Moreover, since demyelination underpins neurodegeneration, miR-214-3p has been described as neuroprotective by reducing the expression of neurodegenerative proteins involved in apoptosis (Varma-Doyle et al., 2021). Contrarily, miR-145-5p might support neurodegeneration as it targets the neuroprotective and anti-inflammatory nuclear receptor subfamily 4 group A member 2 (*NR4A2*) (Liu et al., 2017; Xie et al., 2017). *NR4A2* knockout worsens EAE (Montarolo et al., 2015). *NR4A2* was, however, found upregulated in the motor cortex on brain autopsies of MS patients, correlating positively with neuronal densities (Pansieri et al., 2023).

### 4.8 Limitations of the study

While we observed comparable effects of the investigated microRNAs on the differentiation of CG-4 cells by three different transfection strategies (with microRNA mimics and their inhibitors as well), we acknowledge that (a) microRNA mimics were transfected at supraphysiological levels and that (b) our investigation was limited to an *in vitro* cell line model and verification by other experimental models are warranted. We have, therefore, diligently balanced the interpretation against both the differentiation vehicle control and miR-NC, relying on the statistically significant data, albeit sometimes with small fold changes. Furthermore, we proposed several mRNA targets based on *in silico* and RNA sequencing data and confirmed their downregulation by independent RT-qPCR analyses. However, for only two predicted targets, the effect of the microRNA mimic was significantly antagonized by its microRNA inhibitor. Hence, the interaction of each microRNA with its predicted targets ideally requires validation by a dual luciferase assay. It would be mechanistically informative to delve into the crucial pathways that mediate OPC and OL physiology, such as Notch, Wnt, ERK/MAPK, and Akt/mTOR signaling pathways. Investigating these pathways both *in vitro* and *in vivo* could elucidate the specific effect of each microRNA and its potential mRNA target(s) on OPC differentiation and thereby verify the biological significance of our findings.

## 5 Conclusion

We have screened the effect of several MS-dysregulated microRNAs on the differentiation of CG-4 cells. Herein, we support by independent RT-qPCR analyses and RNA sequencing that miR-33-3p, miR-34c-5p, and miR-124-5p arrest OPC differentiation at a late progenitor stage and miR-145-5p at a premyelinating stage, while only miR-214-3p promotes (initial) OPC differentiation. In this study, we propose a deeply comprehensive investigation of their effect on OPC differentiation. Moreover, for each microRNA, we confirmed the downregulation of several predicted mRNA targets that supposedly support the cell fate changes induced by each microRNA by very distinctive mechanisms, some of which remain unexplored in OPC/OL physiology. Many microRNAs are intricately involved in MS pathogenesis, possibly at different intra- and intercellular levels, of which we currently master only a very limited part. While microRNA-based therapeutic strategies could target these different levels at once, they might also cause undesired on- and off-target effects as a single microRNA potentially targets hundreds of mRNAs and as a single mRNA is finely tuned by several microRNAs. However, further elucidating the role of the microRNAs, the proposed mRNA targets and their respective pathways could pave the way for developing new remyelinating treatment strategies, an unmet need to counteract chronic neurological disability in MS.

## Data availability statement

The RNA sequencing data analyzed for this study are deposited in NCBI’s Gene Expression Omnibus (Edgar et al., 2002), GEO Series accession number GSE244479 (https://www.ncbi.nlm.nih.gov/geo/query/acc.cgi?acc=GSE244479). The other data that support the findings of this study are available from the corresponding author upon reasonable request.

## Ethics statement

Ethical approval was not required for the studies on animals in accordance with the local legislation and institutional requirements because only commercially available established cell lines were used.

## Author contributions

OP: Conceptualization, Data curation, Formal analysis, Funding acquisition, Investigation, Methodology, Project administration, Validation, Visualization, Writing – original draft, Writing – review & editing. PB: Conceptualization, Methodology, Project administration, Validation, Writing – review & editing, Formal analysis. VvP: Conceptualization, Funding acquisition, Supervision, Validation, Writing – review & editing, Methodology, Project administration, Resources.

## Footnotes

^1^ http://www.mirdb.org/index.html

^2^ https://www.targetscan.org/

^3^ http://www.microrna.gr/microT-CDS

^4^ http://mirwalk.umm.uni-heidelberg.de/

^5^ https://genome.crg.eu/CPE
