# Supplementary material for: MicroRNAs dysregulated in multiple sclerosis affect the differentiation of CG-4 cells, an oligodendrocyte progenitor cell line
Source: Front Cell Neurosci. 2024 Feb 29;18:1336439. doi: 10.3389/fncel.2024.1336439 (PMC10937391; doi:10.3389/fncel.2024.1336439)
Supplement: Supplementary file 1 [file Data_Sheet_1.pdf]

Supplementary Material

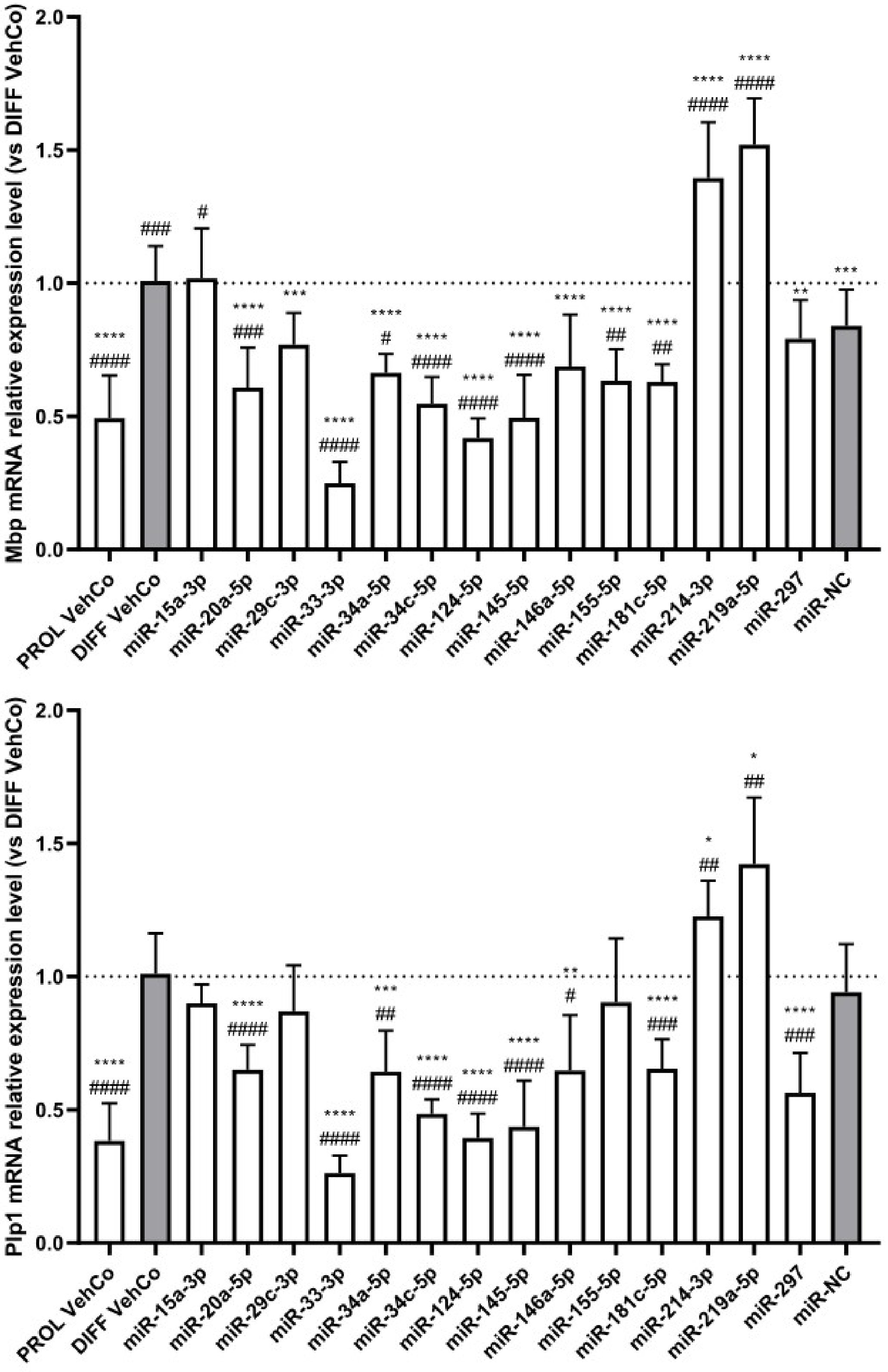

Supplementary figure 1: The effect of 13 microRNA mimics on the differentiation of CG-4 cells in RT-qPCR (screening). The plots show the relative expression (mean with SD of $2^{-\Delta\Delta Ct}$) of the differentiation markers *Mbp* and *Plp1* to the differentiation vehicle control (DIFF VehCo)

resulting from the double transfection of each microRNA mimic (as labelled on the *x*-axis) in CG-4 cells in differentiation culture conditions, as well as the proliferation vehicle control (PROL VehCo). The dotted line represents the mean relative expression of the differentiation vehicle control to which the relative expression ($2^{-\Delta\Delta Ct}$) of each sample was calculated. The adjusted p-value was calculated by Dunnett's multiple comparison test to each control separately (gray bars), i.e. the differentiation vehicle control (*) and the microRNA mimic negative control (miR-NC, #), as recommended following a parametric one-way ANOVA on all conditions: ns for non-significant, */# for $p \leq 0.05$, **/## for $p \leq 0.01$, ***/### for $p \leq 0.001$, ****/#### for $p \leq 0.0001$. Data of 3 experiments for each microRNA with each condition in triplicate. SD, standard deviation.

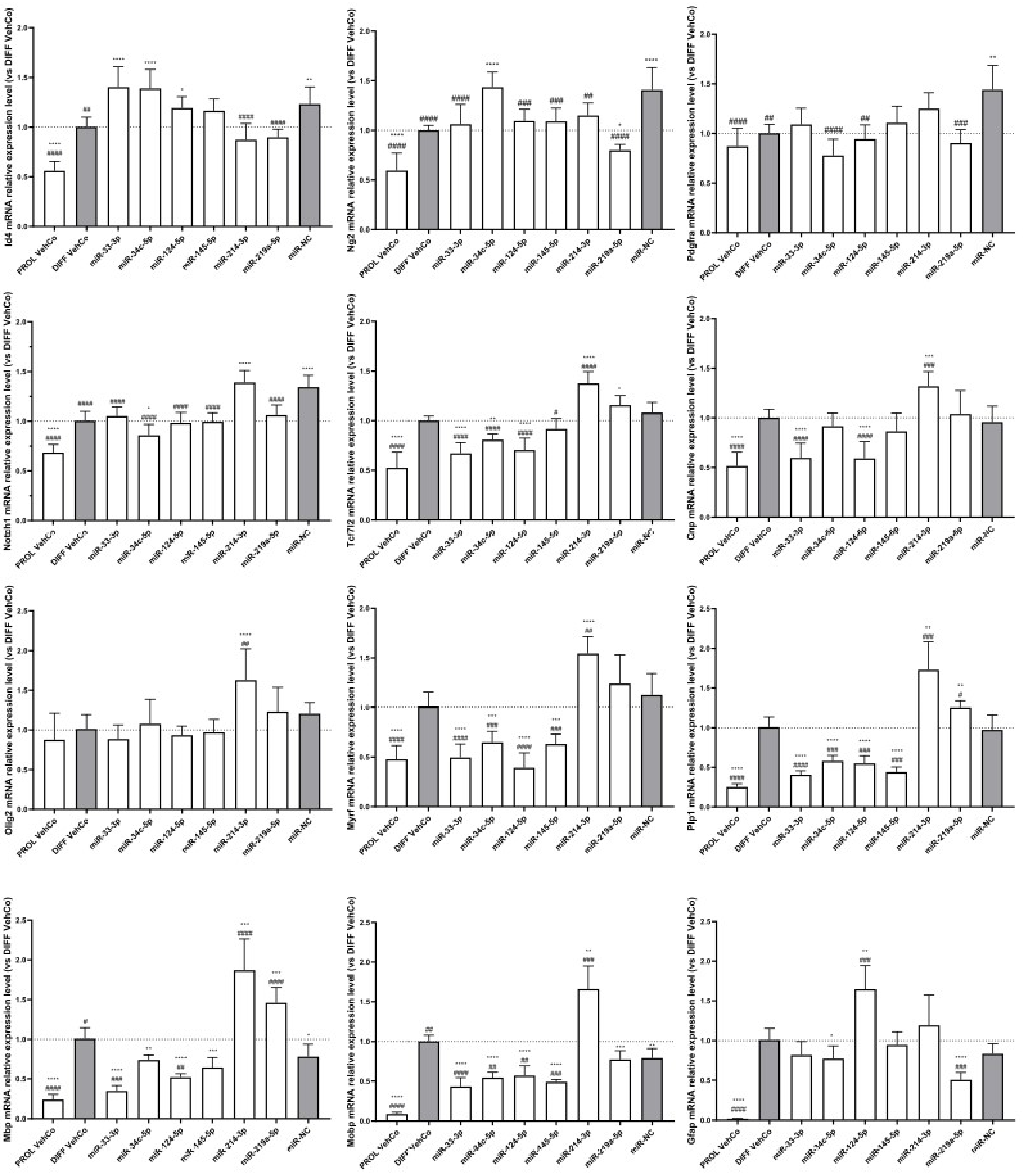

Supplementary figure 2: The effect of 5 microRNA mimics on the differentiation of CG-4 cells in RT-qPCR (confirmation). The plots show the relative expression (mean with SD of $2^{-\Delta\Delta Ct}$) of several OPC, pre-OL, OL and astrocyte markers to the differentiation vehicle control (DIFF VehCo) resulting from the single transfection of each microRNA mimic (as labelled on the *x*-axis) in CG-4 cells in differentiation culture conditions, as well as the proliferation vehicle control (PROL VehCo). The dotted line represents the mean relative expression of the differentiation vehicle control to which the relative expression ($2^{-\Delta\Delta Ct}$) of each sample was calculated. The adjusted p-value was calculated by Dunn's or Dunnett's multiple comparison test to each control separately (gray bars), i.e. the differentiation vehicle control (*) and the microRNA mimic negative control (miR-NC, #), as recommended following a parametric/non-parametric one-way ANOVA on all conditions: ns for non-significant, */# for $p \leq 0.05$, **/##

for $p \leq 0.01$, ***/### for $p \leq 0.001$, ****/#### for $p \leq 0.0001$. Data of 3 experiments with each condition in triplicate. OPC, oligodendrocyte progenitor cells; OL, oligodendrocytes; SD, standard deviation.

A

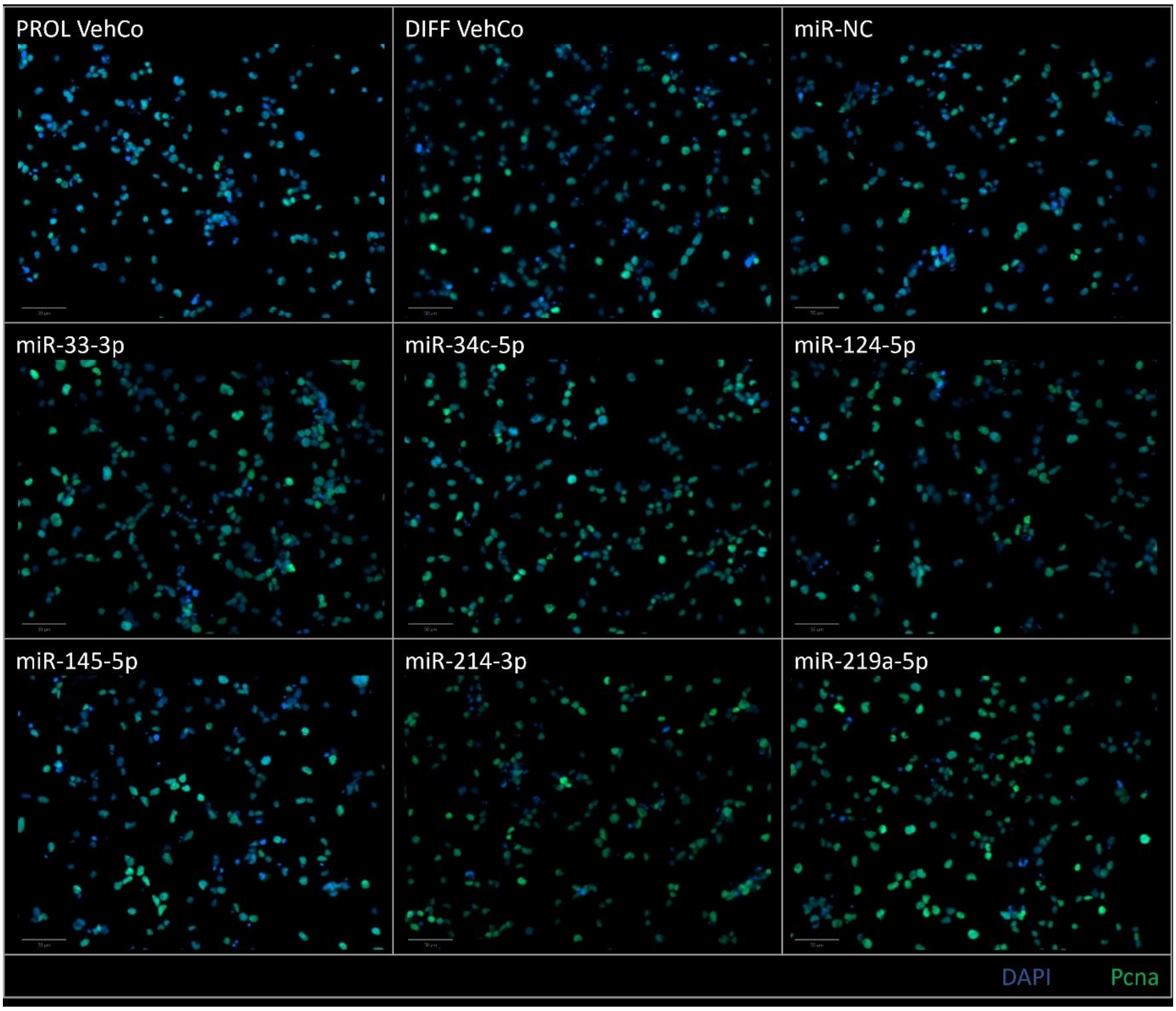
PROL VehCo
DIFF VehCo
miR-NC
miR-33-3p
miR-34c-5p
miR-124-5p
miR-145-5p
miR-214-3p
miR-219a-5p
DAPI
Pcna

B

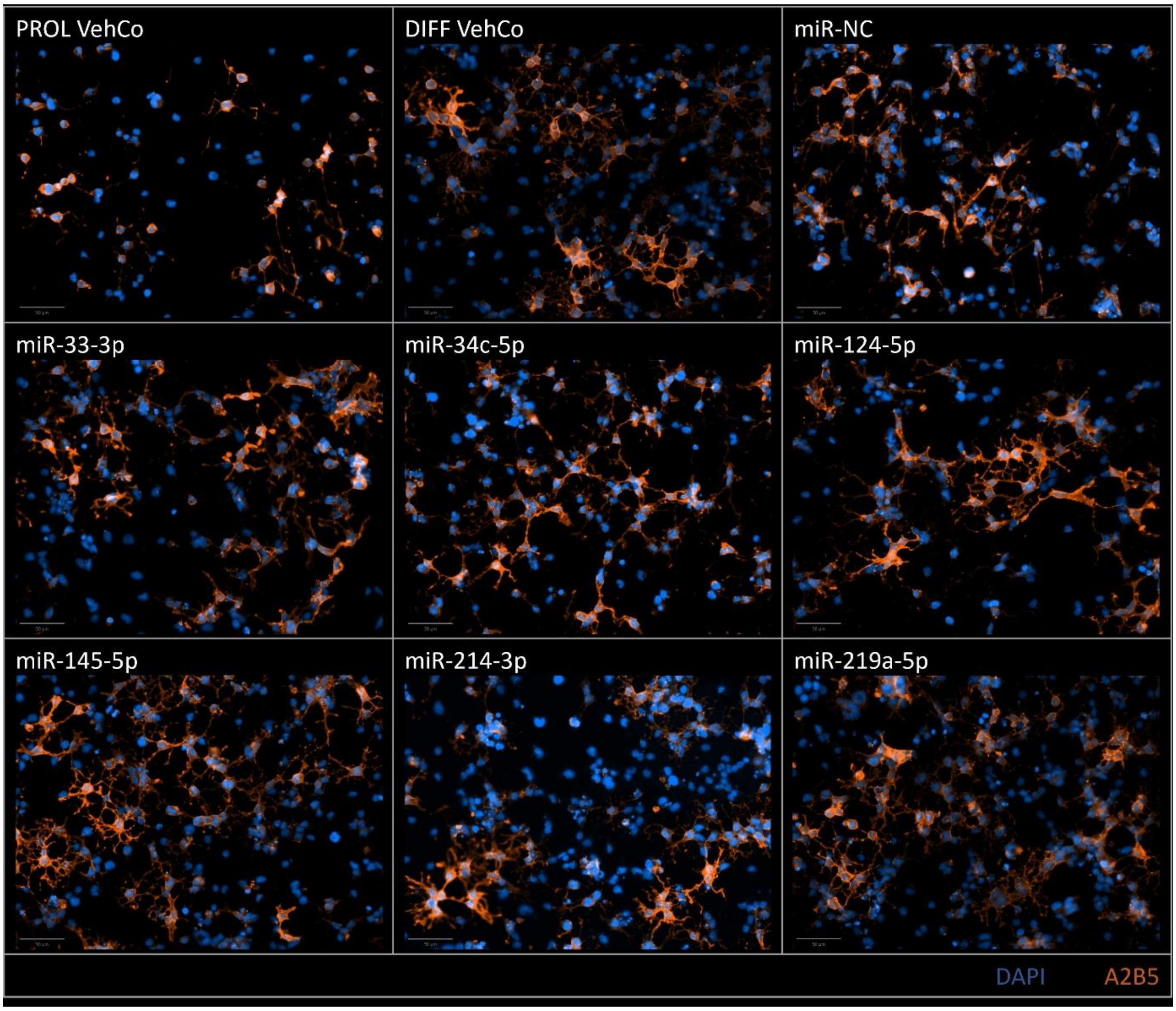
PROL VehCo
DIFF VehCo
miR-NC
miR-33-3p
miR-34c-5p
miR-124-5p
miR-145-5p
miR-214-3p
miR-219a-5p
DAPI
A2B5

C

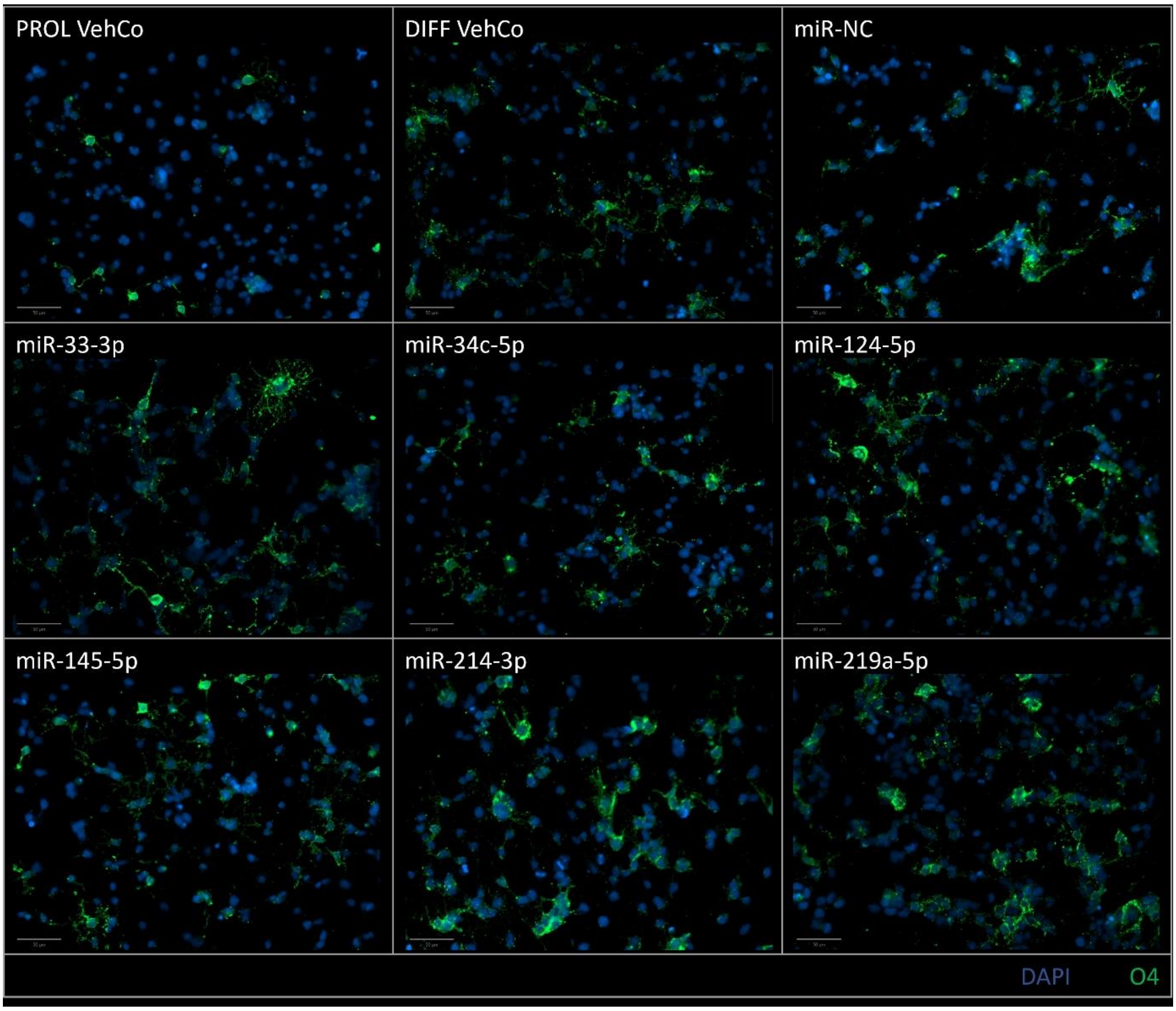
PROL VehCo
DIFF VehCo
miR-NC
miR-33-3p
miR-34c-5p
miR-124-5p
miR-145-5p
miR-214-3p
miR-219a-5p
DAPI
O4

D

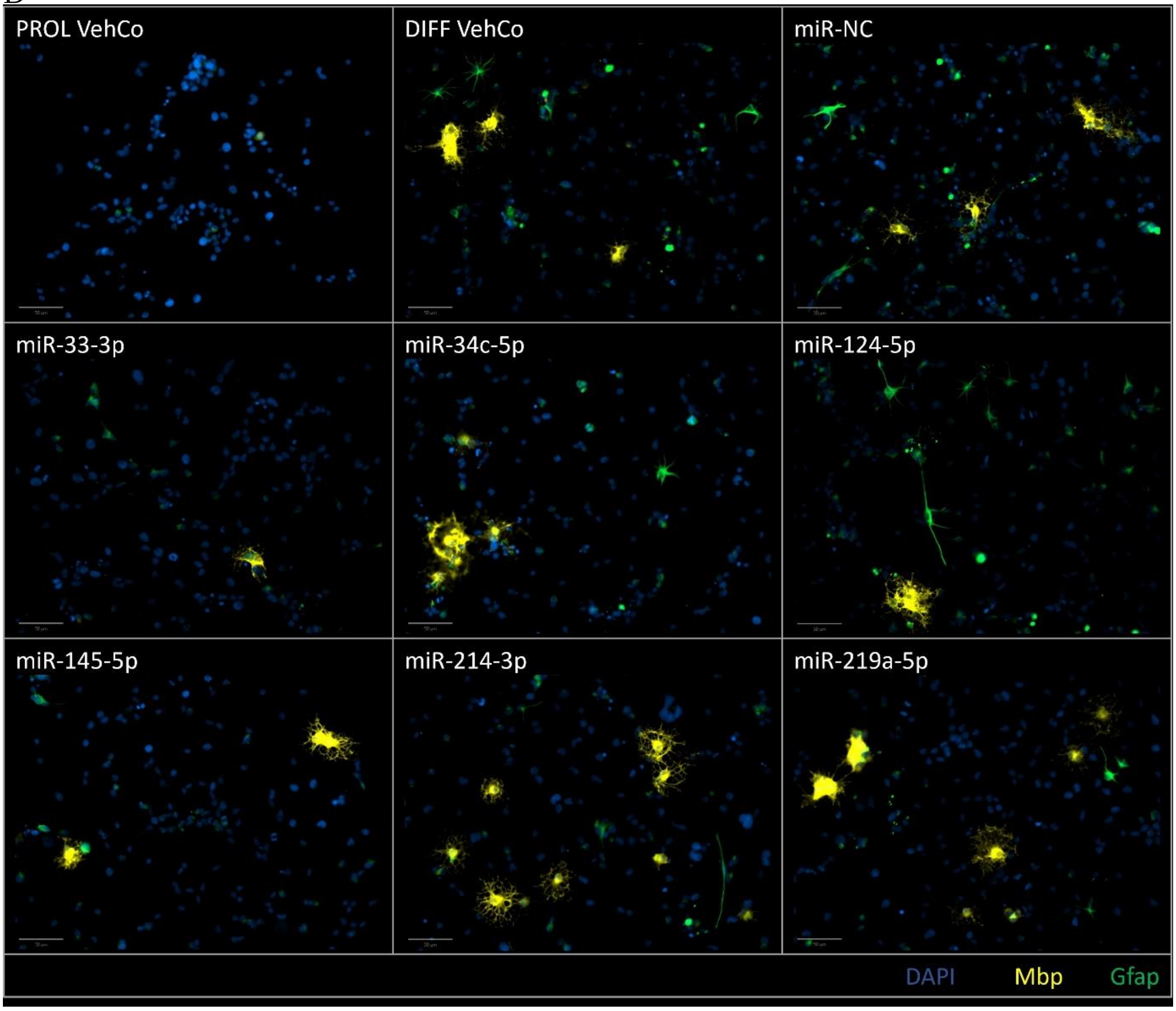

Supplementary figure 3: The effect of 5 microRNA mimics on the proliferation and differentiation of CG-4 cells in immunocytochemistry. These fluorescence micrographs are representative of the staining of (A) proliferation marker Pcna (green), (B) OPC marker A2B5 (orange), (C) late progenitor/early differentiation marker O4 (green), and (D) mature oligodendrocyte marker Mbp (yellow) and astrocyte marker Gfap (green) in CG-4 cells following the single transfection of each microRNA mimic (as labelled in white) in differentiation culture conditions, alongside the proliferation and differentiation vehicle controls (PROL and DIFF VehCo). Of note, Mbp-staining suggests the branching morphology of mature oligodendrocytes. All cell nuclei were counterstained with DAPI (blue). Scale bars = 50 µm. OPC, oligodendrocyte progenitor cells; miR-NC, microRNA negative control.

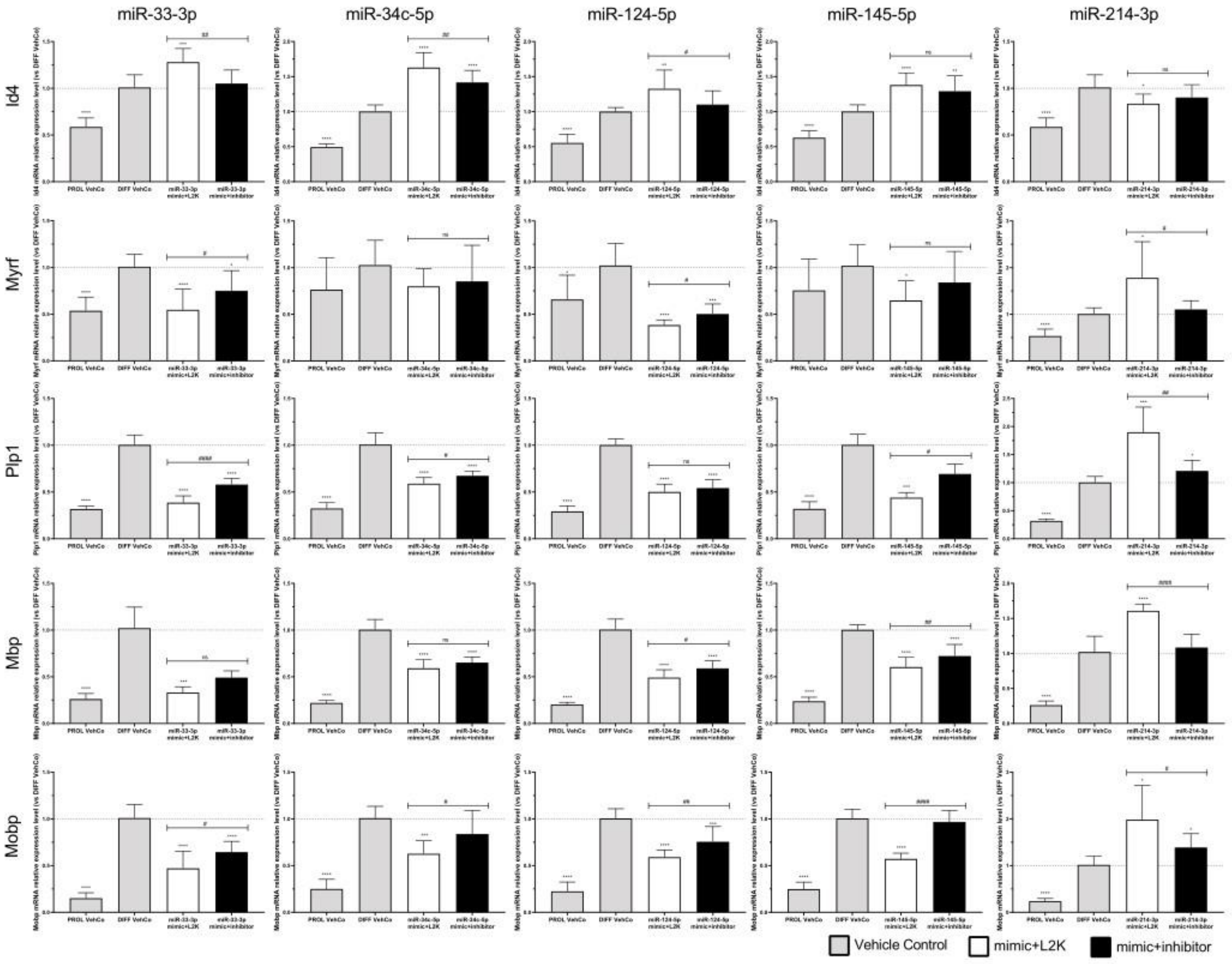
miR-33-3p
miR-34c-5p
miR-124-5p
miR-145-5p
miR-214-3p
Id4
Myrf
Plp1
Mbp
Mobp
Vehicle Control
mimic+L2K
mimic+inhibitor

Supplementary figure 4: The effect of 5 microRNA mimics and their inhibitors on the differentiation of CG-4 cells in RT-qPCR. The plots show the relative expression (mean with SD of $2^{-\Delta\Delta Ct}$) of the OPC marker (*Id4*) and OL markers *(Myrf, Plp1, Mbp,* and *Mobp)* to the differentiation vehicle control (DIFF VehCo, gray bar), resulting from the sequential transfection of each microRNA mimic followed (black bar) or not (white bar) by its inhibitor (as labelled on the *x*-axis) in CG-4 cells in differentiation culture conditions, as well as the proliferation vehicle control (PROL VehCo, gray bar). The dotted line represents the mean relative expression of the differentiation vehicle control to which the relative expression ($2^{-\Delta\Delta Ct}$) of each sample was calculated. The adjusted p-value was calculated by Dunn's or Dunnett's multiple comparison test to the differentiation vehicle control (*) and by Sidak's, Tamhane's T2 or Dunn's multiple comparison test between the microRNA mimic and its inhibitor (#), as recommended following a parametric/non-parametric one-way ANOVA on all conditions: ns for non-significant, */# for $p \leq 0.05$, **/## for $p \leq 0.01$, ***/### for $p \leq 0.001$, ****/#### for $p \leq 0.0001$. Data of 3 experiments for each microRNA with each condition in triplicate. L2K, Lipofectamine™ 2000; SD, standard deviation.

A

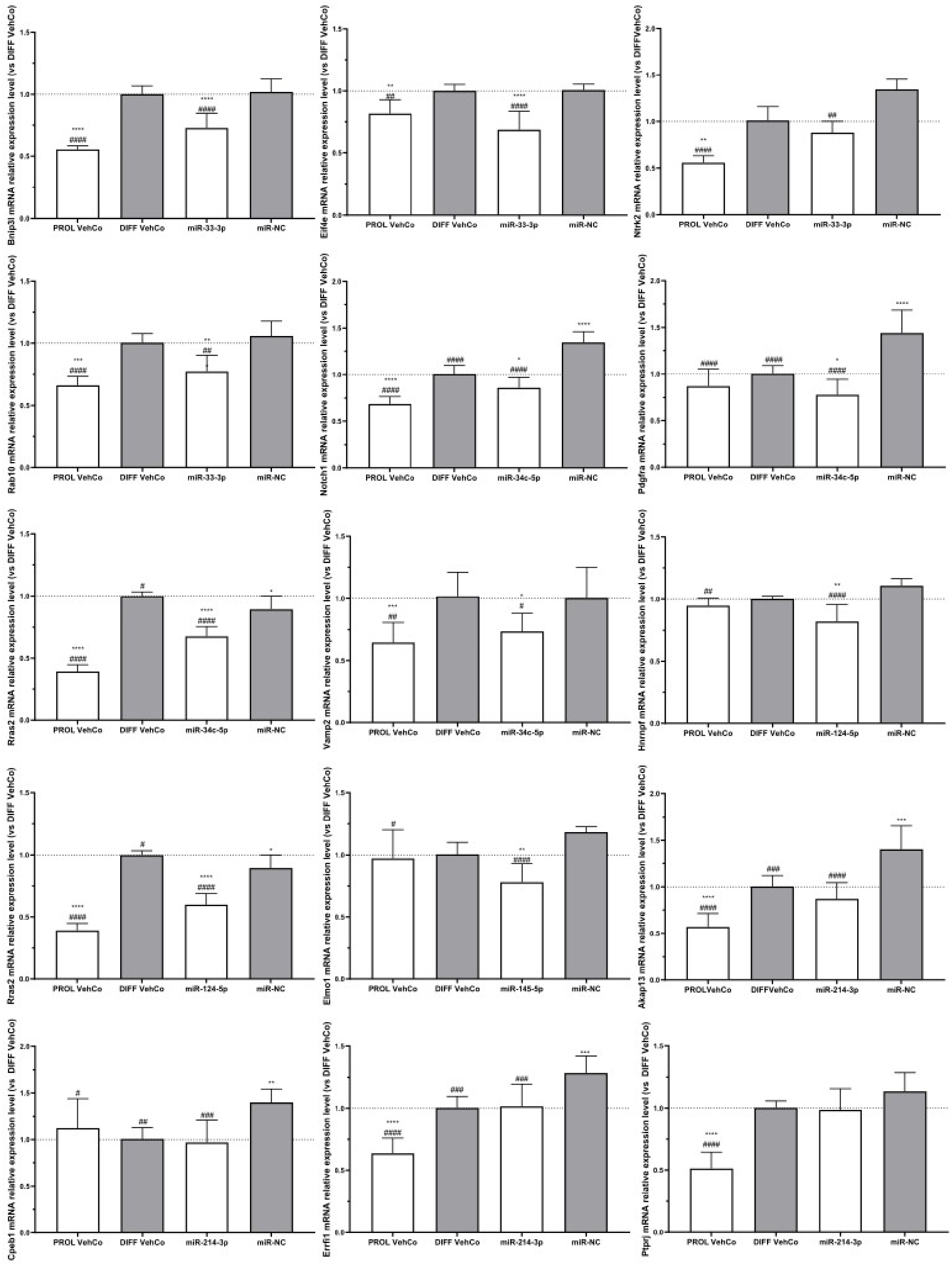
Bnip3l mRNA relative expression level (vs DIFF VehCo)
Eif4e mRNA relative expression level (vs DIFF VehCo)
Ntrk2 mRNA relative expression level (vs DIFFVehCo)
Rab10 mRNA relative expression level (vs DIFF VehCo)
Notch1 mRNA relative expression level (vs DIFF VehCo)
Pdgfra mRNA relative expression level (vs DIFF VehCo)
Rras2 mRNA relative expression level (vs DIFF VehCo)
Vamp2 mRNA relative expression level (vs DIFF VehCo)
Hnrnpf mRNA relative expression level (vs DIFF VehCo)
Rras2 mRNA relative expression level (vs DIFF VehCo)
Elmo1 mRNA relative expression level (vs DIFF VehCo)
Akap13 mRNA relative expression level (vs DIFF VehCo)
Cpeb1 mRNA relative expression level (vs DIFF VehCo)
Errfi1 mRNA relative expression level (vs DIFF VehCo)
Ptprj mRNA relative expression level (vs DIFF VehCo)
PROL VehCo
DIFF VehCo
miR-33-3p
miR-34c-5p
miR-124-5p
miR-145-5p
miR-214-3p
miR-NC

B

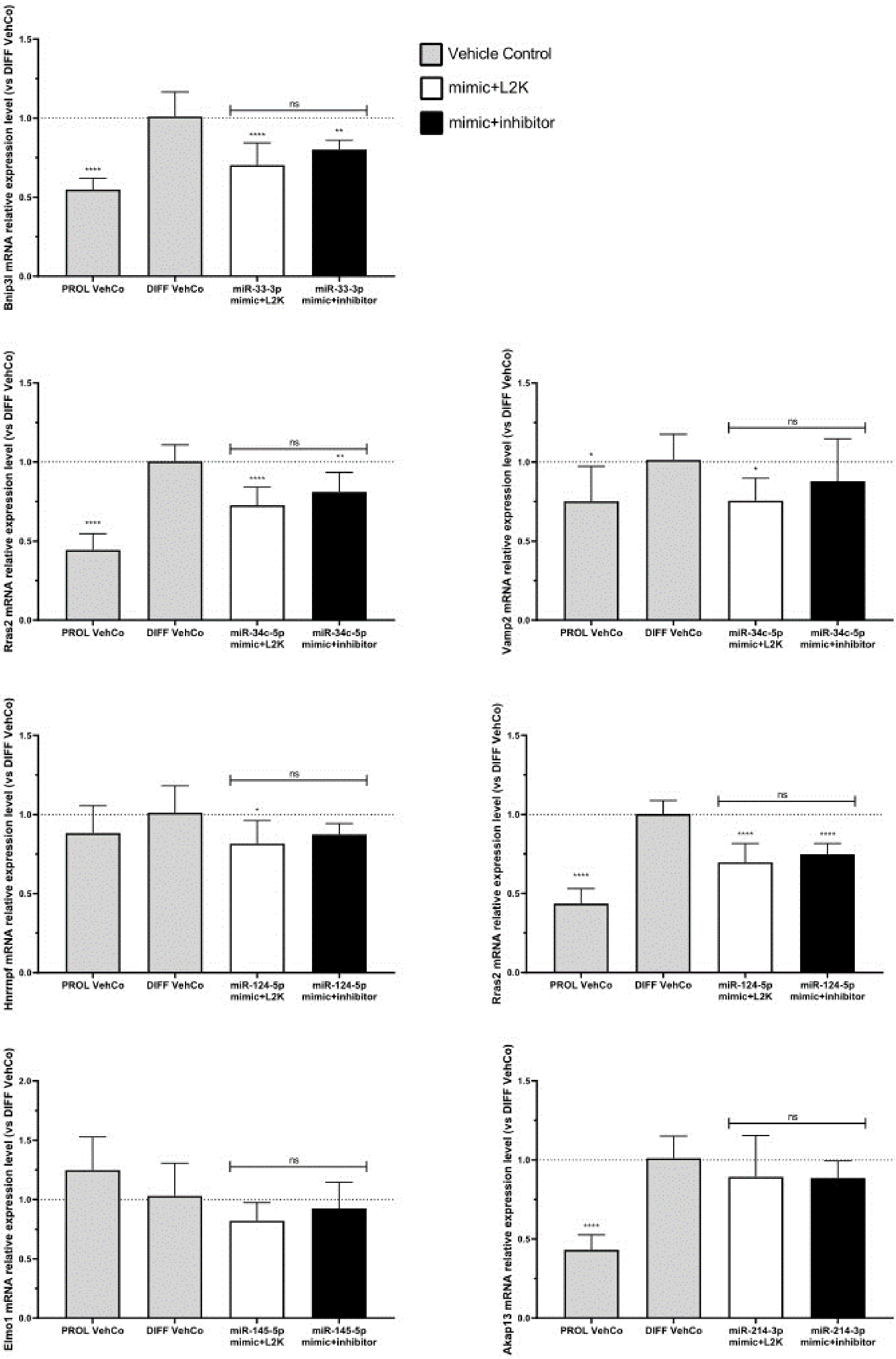
Vehicle Control
mimic+L2K
mimic+inhibitor
Bnip3l mRNA relative expression level (vs DIFF VehCo)
PROL VehCo
DIFF VehCo
miR-33-3p mimic+L2K
miR-33-3p mimic+inhibitor
ns
Rras2 mRNA relative expression level (vs DIFF VehCo)
miR-34c-5p mimic+L2K
miR-34c-5p mimic+inhibitor
Vamp2 mRNA relative expression level (vs DIFF VehCo)
Hnrnpf mRNA relative expression level (vs DIFF VehCo)
miR-124-5p mimic+L2K
miR-124-5p mimic+inhibitor
Elmo1 mRNA relative expression level (vs DIFF VehCo)
miR-145-5p mimic+L2K
miR-145-5p mimic+inhibitor
Akap13 mRNA relative expression level (vs DIFF VehCo)
miR-214-3p mimic+L2K
miR-214-3p mimic+inhibitor

Supplementary figure 5: Potential mRNA targets of each microRNA verified in RT-qPCR by (A) the microRNA mimic and (B) the microRNA mimic and its inhibitor. The plots show the relative expression (mean with SD of $2^{-\Delta\Delta Ct}$) of the potential mRNA targets of each microRNA to the differentiation vehicle control (DIFF VehCo, gray bar), resulting (A) from the single transfection of each microRNA mimic, or (B) from the sequential transfection of each microRNA mimic followed (black bar) or not (white bar) by its inhibitor (as labelled on the *x*-axis) in CG-4 cells in differentiation culture conditions, as well as the proliferation vehicle control (PROL VehCo). The dotted line represents the mean relative expression of the differentiation vehicle control to which the relative expression ($2^{-\Delta\Delta Ct}$) of each sample was calculated. The adjusted p-value was calculated (A) by Dunn's or Dunnett's multiple comparison test to the differentiation vehicle control (*) and to the microRNA negative control mimic (miR-NC, #), and (B) by Dunnett's multiple comparison test to the differentiation vehicle control (*) and by Sidak's or Tamhane's T2 multiple comparison test between the microRNA mimic and its inhibitor (#), as recommended following a parametric/non-parametric one-way ANOVA on all conditions: ns for non-significant, */# for $p \leq 0.05$, **/## for $p \leq 0.01$, ***/### for $p \leq 0.001$, ****/#### for $p \leq 0.0001$. Data of 3 experiments for each microRNA with each condition in triplicate. L2K, Lipofectamine™ 2000; SD, standard deviation.

A

mRNA	Forward primer - sequence 5'-3'	Reverse primer - sequence 5'-3'
rno_Akap13	TGCAGAAATGAACCAGCGGA	GGGACACCAGCTCATACTTCT
rno_Bnip3l	TGTCTCACTTAGTCGAGCCGC	CTCCACCCAGGAACTGTTGA
rno_Cnp	GAGCTTCGACACTTCATTTCTGG	TGGCCTTCCCGTAGTCACAG
rno_Cpeb1	CTCTGGAAGAAGAATCAGGAAGGA	CCAGTGAATCATCCAAAATGGCA
rno_Eif4e	AGATGGCGACTGTGGAACCG	CCAGAGTGCCCACCTGTTCT
rno_Elmo1	TGATGAGAGGAGACAGGAGATGG	GTGCTCGGATGACATGGGTTA
rno_Errfi1	GGGGCAGTCGCAATGAGTT	AGAAGCACATCCGAGGGTTG
rno_Gfap	GGGATGGCGAGGTCATTAAG	TCTGAGGAGGGAGCTTTAGG
rno_Grb2	TCTCCCTGTCAGTCAAGTTTGG	CTTCACCACCCACAGGAAGTAC
rno_Hnrnpf	GCATCTGTGGTGGTTCTTTAAGC	ATTGAGCAGGACCAGGGTAG
rno_Hprt1	GTCATGTCGACCCTCAGTCC	GCAAGTCTTTCAGTCCTGTCC
rno_Id4	CGTTGTGACAAGCGAACTGT	AAAAGTTCCCCGCCCTGTTA
rno_Mbp	GTGGGGGTAAGAGAAACGCA	CGAACACTCCTGTGGAACGA
rno_Mobp	CAGAGCACTTCAGCATCCACT	CTCCTCCTTCTCAATCTGGTCTTC
rno_Myrf	TGCCAACAACATGCGGAAGAAG	GGGTTAGAGGCCCGAACAATGA
rno_Ng2	AGGCCCTCAGGGGAATAGAC	CAGGGCTCCTCTGTGTGAGA
rno_Notch1	TGCGTGGACAAGATCAACGA	GGTCCCCGTGTAACCTTCTG
rno_Ntrk2	CGGGAGCATCTCTCGGTCTA	CCTTTCATGCCAAACTTGGAATG
rno_Olig2	TCAAATCGAATTCACATTCGGAAG	CGTGGATGAGGACACGGTTC
rno_Pdgfra	TCTTATGGCGTTCTGCTCTG	CCATCCTGTATCCGCTCTTG
rno_Plp1	GGCGACTACAAGACCACCAT	AATGACACACCCGCTCCAAA
rno_Ptprj	CCAAGTTAATCCGAGTGGAGA	TCCAATCAGCTTCAGATCCTCA
rno_Rab10	GAAAGGCAAAGGAGAGCAGATTG	TCAGCTAATGTGAGGAACGCC
rno_Rras2	GACCATCAGAGACAGGTAACGC	AACTCGGACAAGTTCGTGGAA
rno_Sox10	AGGTTGCTGAACGAGAGTGAC	CGCCGAGGTTGGTACTTGTAG
rno_Tcf7l2	ATGGTTAGTACCACAGCAAGGT	AGAGTGTGATGGGGAGGGAC
rno_Vamp2	CCCTGCACCTCCTCCAAATC	GATGTCCACCACCTCATCCAC

B

miRCURY LNA miRNA (Qiagen)	Mimic (Cat No. 339173)	Inhibitor (Cat. No. 339121)	PCR Assay (Cat. No. 339306)		miRBase
microRNA ID		GeneGlobe ID		sequence 5'-3'	MIMAT ID
hsa-miR-15a-3p	YM00471094	NA	NA	CAGGCCAUAUUGUGCUGCCUCA	MIMAT0004488
hsa-miR-20a-5p	YM00472205	NA	NA	UAAAGUGCUUAUAGUGCAGGUAG	MIMAT0000075
hsa-miR-29c-3p	YM00470481	NA	NA	UAGCACCAUUUGAAAUCGGUUA	MIMAT0000681
rno-miR-33-3p	YM00471597	YI04100217	YP02113481	CAAUGUUUCCACAGUGCAUCA	MIMAT0017104
hsa-miR-34a-5p	YM00473212	NA	NA	UGGCAGUGUCUUAGCUGGUUGU	MIMAT0000255
hsa-miR-34c-5p	YM00472810	YI04101981	YP00205659	AGGCAGUGUAGUUAGCUGAUUGC	MIMAT0000686
hsa-miR-103a-3p	NA	NA	YP00204063	AGCAGCAUUGUACAGGGCUAUGA	MIMAT0000101
hsa-miR-124-5p	YM00470547	YI04101111	YP00204266	CGUGUUCACAGCGGACCUUGAU	MIMAT0004591
hsa-miR-145-5p	YM00470014	YI04102423	YP00204483	GUCCAGUUUUCCCAGGAAUCCCU	MIMAT0000437
hsa-miR-146a-5p	YM00472124	NA	NA	UGAGAACUGAAUUCCAUGGGUU	MIMAT0000449
mmu-miR-155-5p	YM00470919	NA	NA	UUAAUGCUAAUUGUGAUAGGGGU	MIMAT0000165
hsa-miR-181c-5p	YM00471280	NA	NA	AACAUUCAACCUGUCGGUGAGU	MIMAT0000258
rno-miR-214-3p	YM00472862	YI04105003	YP00205512	ACAGCAGGCACAGACAGGCAG	MIMAT0000885
hsa-miR-219a-5p	YM00470876	NA	NA	UGAUUGUCCAAACGCAAUUCU	MIMAT0000276
rno-miR-297	YM00470075	NA	NA	AUGUAUGUGUGCAUGUAUGCAUG	MIMAT0000899
Negative Control	YM00479902	NA	NA	UCACCGGGUGUAAAUCAGCUUG	NA

Supplementary table 1: Sequences of mRNA primers, microRNA mimics and microRNA primers. (A) List of forward and reverse primers of the investigated mRNAs. (B) List of microRNA mimics, inhibitors, and PCR Assay primers (miRCURY LNA miRNA, Qiagen) with corresponding GeneGlobe ID (Qiagen database), sequence and MIMAT ID (miRbase database). Interspecies compatibility was strictly verified. hsa, *Homo sapiens*; mmu, *Mus musculus*; rno, *Rattus norvegicus*; NA, not applicable.

A

	vs. DIFF VehCo		vs. miR-NC	
mRNA	padj	Log2(FC)	padj	Log2(FC)
**miR-33-3p targets**				
Ntrk2	2.41E-08	-0.18	2.73E-14	-0.34
Rab10	4.28E-05	-0.15	2.96E-06	-0.19
Eif4e	5.57E-21	-0.36	6.28E-06	-0.25
Bnip3l	3.78E-10	-0.22	1.96E-05	-0.18
Stim2	1.69E-02	-0.14	4.45E-03	-0.20
Fam168b	5.15E-03	-0.10	2.11E-03	-0.13
Sec63	1.37E-08	-0.23	1.25E-12	-0.34
**miR-34c-5p targets**				
Vamp2*	5.08E-28	-0.43	8.74E-17	-0.38
Vdr*	1.05E-24	-0.59	6.85E-13	-0.53
Rras2*	1.23E-03	-0.30	ns	
**miR-124-5p targets**				
Rras2*	8.31E-16	-0.67	2.93E-02	-0.28
Cdon*	5.43E-07	-0.55	1.86E-05	-0.60
Hnrnpab*	1.76E-28	-0.44	2.57E-21	-0.50
Hnrnpf	1.91E-04	-0.11	2.24E-02	-0.10
**miR-145-5p targets**				
Myrf	2.96E-76	-0.77	6.53E-32	-0.56
Snx27	4.45E-09	-0.22	1.59E-03	-0.18
**miR-214-3p targets**				
Id4^{+}	3.05E-04	-0.28	1.24E-06	-0.50
Errfi1	2.71E-04	-0.26	1.17E-05	-0.37
Grb2*	1.19E-04	-0.14	7.59E-06	-0.22
Fgfr1^{+}	3.10E-06	-0.15	9.30E-11	-0.28

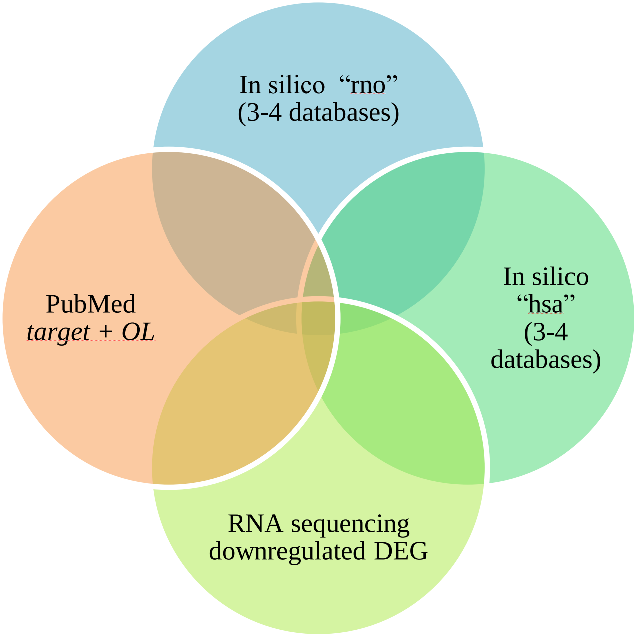

B

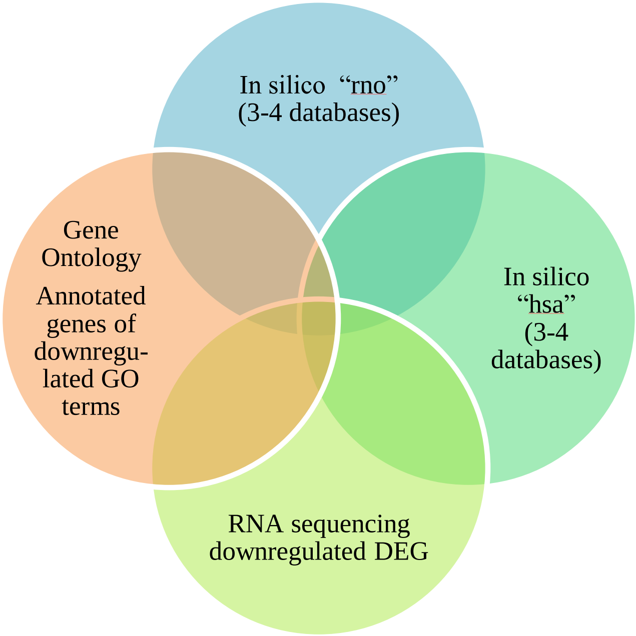

	vs. DIFF VehCo		vs. miR-NC		Gene Ontology term (Biological Process)
mRNA	padj	Log2(FC)	padj	Log2(FC)	
**miR-33-3p targets**					
Calm2	4.68E-07	-0.16	1.36E-02	-0.11	cell division
Etnk1	4.15E-02	-0.08	4.01E-02	-0.11	phospholipid biosynthetic process
Far1	2.67E-21	-0.40	6.05E-12	-0.40	phospholipid biosynthetic process<br>long-chain fatty-acyl-CoA metabolic process
Ndel1	2.75E-04	-0.19	4.09E-03	-0.20	chromosome segregation
Ntrk2	2.41E-08	-0.18	2.73E-14	-0.34	myelination<br>axon ensheathment
Rab10	4.28E-05	-0.15	2.96E-06	-0.19	cell division
Slc1a3	1.51E-02	-0.12	3.91E-13	-0.41	generation of precursor metabolites and energy
Ube2j1	2.79E-02	-0.13	6.40E-05	-0.27	regulation of protein catabolic process
Ube2n	8.00E-30	-0.46	1.32E-10	-0.35	DNA repair
**miR-34c-5p targets**					
Ago4	4.62E-07	-0.46	2.69E-04	-0.44	ribonucleoprotein complex assembly
Atg4b	3.59E-03	-0.13	2.92E-02	-0.13	regulation of protein catabolic process
Dixdc1	7.73E-03	-0.14	5.39E-03	-0.17	Wnt signaling pathway<br>stress-activated MAPK cascade<br>axonogenesis

Dll1	3.24E-04	-0.29	5.77E-10	-0.54	astrocyte differentiation<br>glial cell differentiation
Erc1	1.09E-21	-0.31	4.33E-02	-0.11	synapse organization
Erlin1					regulation of lipid biosynthetic process<br>cholesterol metabolic process
Map1a	6.31E-13	-0.23	6.03E-05	-0.16	regulation of protein catabolic process<br>axonogenesis
Notch1	1.54E-28	-0.32	6.11E-21	-0.34	Wnt signaling pathway<br>Ras protein signal transduction<br>astrocyte differentiation<br>oligodendrocyte differentiation<br>negative regulation of gliogenesis<br>axonogenesis
Pdgfra	7.60E-47	-0.34	7.92E-60	-0.50	positive regulation of kinase activity<br>regulation of phospholipid metabolic process
Pip5k1a	1.16E-09	-0.26	7.49E-08	-0.29	glycerolipid biosynthetic process
Ppp2r5a	8.38E-06	-0.23	9.80E-05	-0.29	regulation of lipid metabolic process
Synj1	1.67E-02	-0.14	6.32E-04	-0.22	regulation of gliogenesis
Vcl	2.86E-06	-0.37	4.27E-07	-0.47	axonogenesis
**miR-124-5p targets**					
Atxn2<br>Hnrnpf	1.42E-04<br>1.91E-04	-0.17<br>-0.11	5.32E-03<br>2.24E-02	-0.17<br>-0.10	ribonucleoprotein complex biogenesis<br>mRNA processing<br>RNA splicing
**miR-145-5p targets**					
Adpgk	1.75E-02	-0.15	2.49E-02	-0.17	pyruvate metabolic process
Arl6ip5	7.97E-06	-0.19	9.17E-03	-0.15	stress-activated protein kinase signaling cascade
Elmo1	2.10E-06	-0.29	5.30E-09	-0.40	Ras protein signal transduction
Map4k4	3.37E-13	-0.20	5.96E-03	-0.13	Ras protein signal transduction<br>stress-activated protein kinase signaling cascade
Myrf	2.96E-76	-0.77	6.53E-32	-0.56	oligodendrocyte differentiation<br>gliogenesis<br>myelination<br>axon ensheathment
Rtkn	7.52E-16	-0.35	3.17E-14	-0.42	Ras protein signal transduction<br>Rho protein signal transduction<br>cell division
**miR-214-3p targets**					
Akap13	4.54E-26	-0.35	9.44E-30	-0.49	positive regulation of kinase activity
Atg13	1.97E-03	-0.18	2.81E-02	-0.17	autophagy
Atp2a2	1.25E-56	-0.41	9.92E-28	-0.38	autophagy

Cdip1	2.05E-06	-0.23	2.09E-02	-0.16	intrinsic apoptotic signaling pathway
Cpeb1	3.74E-03	-0.83	6.65E-04	-1.13	cytoplasmic translation
Csf1	3.21E-03	-0.26	5.09E-06	-0.45	positive regulation of kinase activity<br>gliogenesis
Errfi1	2.71E-04	-0.26	1.17E-05	-0.37	response to platelet-derived growth factor<br>negative regulation of ERBB signaling pathway
Gpd1	2.23E-14	-1.10	1.72E-17	-1.35	glycerolipid metabolic process<br>generation of precursor metabolites and energy<br>pyruvate biosynthetic process<br>carbohydrate catabolic process<br>ATP biosynthetic process
Ifnar1	1.18E-08	-0.23	4.03E-11	-0.32	generation of precursor metabolites and energy
Mtmr3	6.38E-03	-0.11	2.89E-02	-0.12	phospholipid metabolic process<br>glycerolipid metabolic process<br>autophagy
Mtmr4	5.09E-03	-0.13	1.23E-02	-0.15	phospholipid metabolic process<br>glycerolipid metabolic process<br>autophagy
Plec	1.18E-26	-0.38	8.89E-08	-0.26	glial cell differentiation<br>myelin maintenance<br>generation of precursor metabolites and energy
Ptprj	3.88E-15	-0.29	2.55E-17	-0.39	insulin receptor signaling pathway<br>negative regulation of ERBB signaling pathway<br>glial cell differentiation

Supplementary table 2: Potential mRNA targets of each microRNA. The potential mRNA targets were predicted by *in silico* analysis, by their downregulation in RNA sequencing and by (A) a Pubmed search of their involvement in OPC/OL physiology or (B) the gene annotation to downregulated Gene Ontology terms of interest as illustrated by the Venn diagrams. We selected the targets predicted by 3-4 databases in both rats and humans, except for (*) only in rats and ($^{+}$) only in humans and predicted by 0-2 databases in the other species. Each table contains the fold change (FC, expressed as logarithm base 2 [Log2(FC)] of the gene expression value of the potential mRNA targets to the differentiation vehicle control (DIFF VehCo) and to the microRNA negative control mimic (miR-NC), highlighted by a blue-color gradient [dark blue for a bigger negative fold change, to light blue for a smaller negative fold change]), as well as the adjusted p-value (padj) in RNA sequencing. DEG, differentially expressed genes; GO, Gene Ontology; hsa, *Homo sapiens*; ns, non-significant; OPC, oligodendrocyte progenitor cells; OL, oligodendrocytes; rno, *Rattus norvegicus*.

Furthermore, we will briefly highlight other targets we did not investigate, but that might deserve further attention, namely Fyn related Src family tyrosine kinase (*Frk*) and vitamin D receptor (*Vdr*) (mir-34c-5p), as well as fibroblast growth factor receptor 1 (*Fgfr1)* (miR-214-3p). *Frk* (miR-34c-5p) has not been described in OPCs, but it is related to Fyn tyrosine kinase. Fyn tyrosine kinase regulates morphological OPC differentiation by activation of Rac1 and inactivation of RhoA, involved in stress fiber formation and actin polymerization, and allows *Mbp* translation in RNA transport granules by phosphorylating Hnrnpa2/Hnrnpf (Liang et al., 2004; White et al., 2012; Pedraza et al., 2014) *Vdr* (miR-34c-5p) was only predicted by one database in humans. However, vitamin D treatment, both *in vitro* and *in vivo,* as well as Vdr signaling induce OPC differentiation (de la Fuente et al., 2015; Gomez-Pinedo et al., 2020; Mengozzi et al., 2020; Li et al., 2022). Herein, vitamin D inhibits OPC proliferation by downregulating Myc proto-oncogene BHLH transcription factor, and upregulates Ras protein signal transduction, that has been linked to OPC differentiation (Mengozzi et al., 2020; Li et al., 2022). Finally, *Fgfr1*, a target of miR-214-3p predicted in humans but not in rats, is involved in OPC specification and proliferation (Baron et al., 2000; Furusho et al., 2011).

References

Baron, W., Metz, B., Bansal, R., Hoekstra, D., and de Vries, H. (2000). PDGF and FGF-2 Signaling in Oligodendrocyte Progenitor Cells: Regulation of Proliferation and Differentiation by Multiple Intracellular Signaling Pathways. *Molecular and Cellular Neuroscience* 15, 314–329. doi: 10.1006/mcne.1999.0827.

de la Fuente, A. G., Errea, O., van Wijngaarden, P., Gonzalez, G. A., Kerninon, C., Jarjour, A. A., et al. (2015). Vitamin D receptor–retinoid X receptor heterodimer signaling regulates oligodendrocyte progenitor cell differentiation. *J Cell Biol* 211, 975–985. doi: 10.1083/jcb.201505119.

Furusho, M., Kaga, Y., Ishii, A., Hébert, J. M., and Bansal, R. (2011). Fibroblast Growth Factor Signaling Is Required for the Generation of Oligodendrocyte Progenitors from the Embryonic Forebrain. *J. Neurosci.* 31, 5055–5066. doi: 10.1523/JNEUROSCI.4800-10.2011.

Gomez-Pinedo, U., Cuevas, J. A., Benito-Martín, M. S., Moreno-Jiménez, L., Esteban-Garcia, N., Torre-Fuentes, L., et al. (2020). Vitamin D increases remyelination by promoting oligodendrocyte lineage differentiation. *Brain Behav* 10, e01498. doi: 10.1002/brb3.1498.

Li, N., Yao, M., Liu, J., Zhu, Z., Lam, T.-L., Zhang, P., et al. (2022). Vitamin D Promotes Remyelination by Suppressing c-Myc and Inducing Oligodendrocyte Precursor Cell Differentiation after Traumatic Spinal Cord Injury. *Int J Biol Sci* 18, 5391–5404. doi: 10.7150/ijbs.73673.

Liang, X., Draghi, N. A., and Resh, M. D. (2004). Signaling from integrins to Fyn to Rho family GTPases regulates morphologic differentiation of oligodendrocytes. *J Neurosci* 24, 7140–7149. doi: 10.1523/JNEUROSCI.5319-03.2004.

Mengozzi, M., Hesketh, A., Bucca, G., Ghezzi, P., and Smith, C. P. (2020). Vitamins D3 and D2 have marked but different global effects on gene expression in a rat oligodendrocyte precursor cell line. *Mol Med* 26, 32. doi: 10.1186/s10020-020-00153-7.

Pedraza, C. E., Taylor, C., Pereira, A., Seng, M., Tham, C.-S., Izrael, M., et al. (2014). Induction of oligodendrocyte differentiation and in vitro myelination by inhibition of rho-associated kinase. *ASN Neuro* 6, 1759091414538134. doi: 10.1177/1759091414538134.

White, R., Gonsior, C., Bauer, N. M., Krämer-Albers, E.-M., Luhmann, H. J., and Trotter, J. (2012). Heterogeneous Nuclear Ribonucleoprotein (hnRNP) F Is a Novel Component of Oligodendroglial RNA Transport Granules Contributing to Regulation of Myelin Basic Protein (MBP) Synthesis *. *Journal of Biological Chemistry* 287, 1742–1754. doi: 10.1074/jbc.M111.235010.
